# Sheep and Goat Genome Engineering: From Random Transgenesis to the CRISPR Era

**DOI:** 10.3389/fgene.2019.00750

**Published:** 2019-09-03

**Authors:** Peter Kalds, Shiwei Zhou, Bei Cai, Jiao Liu, Ying Wang, Bjoern Petersen, Tad Sonstegard, Xiaolong Wang, Yulin Chen

**Affiliations:** ^1^Key Laboratory of Animal Genetics, Breeding and Reproduction of Shaanxi Province, College of Animal Science and Technology, Northwest A&F University, Yangling, China; ^2^Department of Animal and Poultry Production, Faculty of Environmental Agricultural Sciences, Arish University, El-Arish, Egypt; ^3^Institute of Farm Animal Genetics, Friedrich-Loeffler-Institut, Neustadt, Germany; ^4^Recombinetics, Saint Paul, MN, United States

**Keywords:** sheep, goats, farm animals, genetic modification, genome engineering, gene editing, CRISPR/Cas9

## Abstract

Sheep and goats are valuable livestock species that have been raised for their production of meat, milk, fiber, and other by-products. Due to their suitable size, short gestation period, and abundant secretion of milk, sheep and goats have become important model animals in agricultural, pharmaceutical, and biomedical research. Genome engineering has been widely applied to sheep and goat research. Pronuclear injection and somatic cell nuclear transfer represent the two primary procedures for the generation of genetically modified sheep and goats. Further assisted tools have emerged to enhance the efficiency of genetic modification and to simplify the generation of genetically modified founders. These tools include sperm-mediated gene transfer, viral vectors, RNA interference, recombinases, transposons, and endonucleases. Of these tools, the four classes of site-specific endonucleases (meganucleases, ZFNs, TALENs, and CRISPRs) have attracted wide attention due to their DNA double-strand break-inducing role, which enable desired DNA modifications based on the stimulation of native cellular DNA repair mechanisms. Currently, CRISPR systems dominate the field of genome editing. Gene-edited sheep and goats, generated using these tools, provide valuable models for investigations on gene functions, improving animal breeding, producing pharmaceuticals in milk, improving animal disease resistance, recapitulating human diseases, and providing hosts for the growth of human organs. In addition, more promising derivative tools of CRISPR systems have emerged such as base editors which enable the induction of single-base alterations without any requirements for homology-directed repair or DNA donor. These precise editors are helpful for revealing desirable phenotypes and correcting genetic diseases controlled by single bases. This review highlights the advances of genome engineering in sheep and goats over the past four decades with particular emphasis on the application of CRISPR/Cas9 systems.

## Introduction

**Figure 1:**
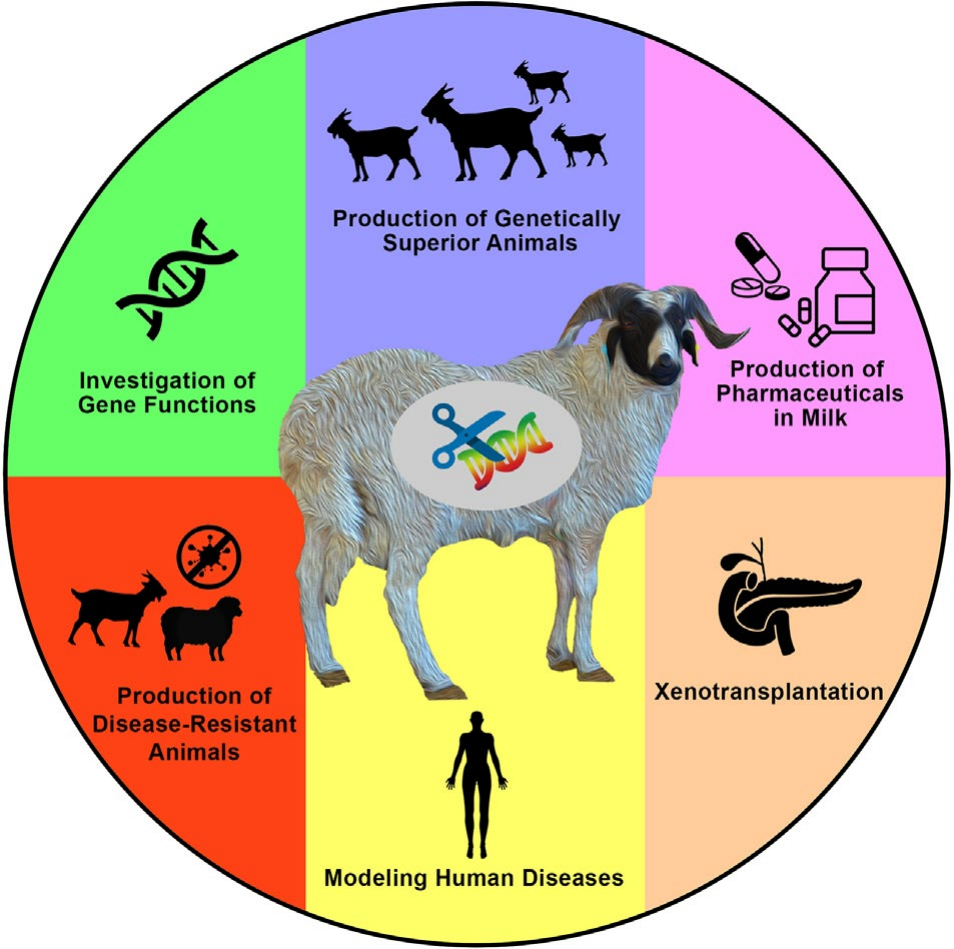
Applications and aims of genome engineering in sheep and goats. Genome engineering has been applied in both sheep and goats (or generally in farm animals) for various purposes such as to investigate the biological and functional roles of genes, to introduce novel economically important traits for agricultural purposes, to produce valuable proteins in milk, to produce animals that are resistant to epidemic diseases, to model human diseases, and to produce hosts for the growth of human organs for xenotransplantation research, among other valuable purposes that mainly aim to increase human knowledge, as well as human and animal health and welfare.

Generating new and variable phenotypes *via* direct alteration of DNA sequences is an interesting idea that has sparked the curiosity of a wide spectrum of researchers over the past few decades. Based on significant efforts, tremendous advances have been achieved in animal genetics and reproductive physiology. These have enabled what is now known as the genome-editing revolution that can be applied to generate gene-edited animals including sheep and goats for various purposes (**Figure 1**).

**Figure 2:**
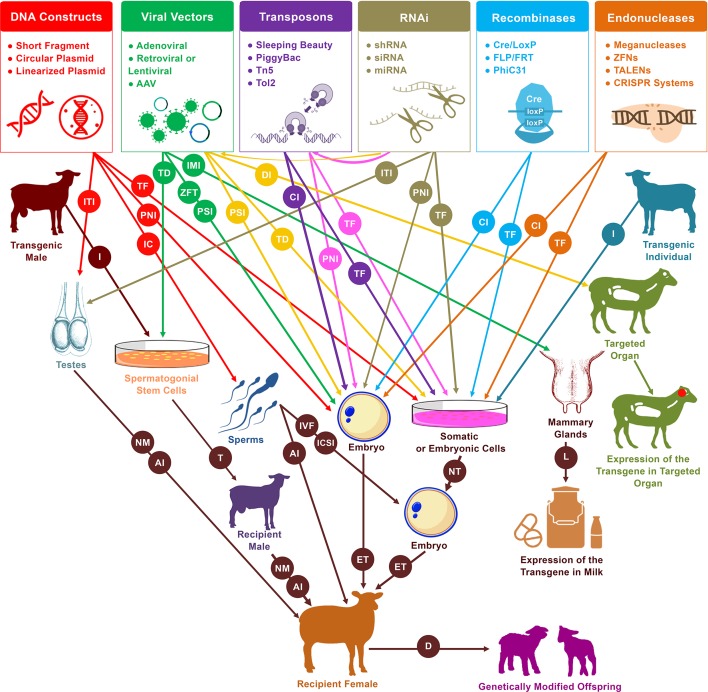
Schematic representation of practical and likely pathways of genetic modification in sheep and goats. Pronuclear injection (PNI) and nuclear transfer (NT) are the two primary procedures for the generation of live founders with desired genetic modifications. In addition to these two approaches, several new tools have emerged that increase the efficiency and simplify the process of mediating genetic modification. These tools include sperm-mediated gene transfer (SMGT), viral vectors, recombinases, transposons, RNA interference (RNAi), and endonucleases. These have served to mediate manipulations in a variety of cells and organs, including somatic cells, embryonic cells, embryos, spermatozoa, spermatogonial stem cells (SSCs), testes, mammary glands, and other targeted organs. Different procedures are involved in the delivery of DNA constructs as well as the various enzymes and systems that induce genetic modification events within genomes. PNI, cytoplasmic injection (CI), perivitelline space injection (PSI), and zona-free transduction (ZFT) have been used for the delivery to embryos, transfection (TF), and transduction (TD) for the delivery to cells, incubation (IC) for the delivery to spermatozoa, intratesticular injection (ITI) for the delivery to testes, intramammary injection (IMI) for the delivery to mammary glands, and direct injection (DI) for the delivery to targeted organs (mainly for medical purposes). *In vitro* fertilization (IVF), intracytoplasmic sperm injection (ICSI), artificial insemination (AI) or even natural mating (NM) have been used for the delivery of transgenic sperms that resulted from incubation treatment, male germ cell transplantation, or intratesticular injection. In the diagram, from left to right, red arrows indicate the uses of DNA constructs for mediating DNA modification, green arrows indicate the uses of viral vectors, yellow arrows indicate the uses of RNAi molecules via delivery by viral vectors, purple arrows indicate the uses of transposons, pink arrows indicate the uses of RNAi molecules via integration by transposons, tan arrows indicate the independent uses of RNAi molecules, light blue arrows indicate the uses of recombinases, and orange arrows indicate the uses of endonucleases. Isolation (I) of spermatogonial stem cells from transgenic males can be used via transplantation (T) into infertile males to generate donor-derived spermatogenesis, which can then be used to generate transgenic founders. Furthermore, isolation of cells from transgenic individuals can also be used by nuclear transfer (NT) to generate transgenic progeny. Other abbreviations used in the diagram include embryo transfer (ET), lactation (L), and delivery (D).

About 40 years ago, a set of basic techniques were applied to sheep embryos with the desire to generate identical twins, multiplets, and chimeras. The further development of these tools has led to the generation of identical individuals by embryo splitting (Willadsen, 1979), chimeras by aggregating embryonic cells (Fehilly et al., 1984b), and even the first cloned sheep prior to the famous *Dolly* from undifferentiated embryonic cells (Willadsen, 1986). During that time, in 1985, the first report about the generation of transgenic farm animals (including sheep) *via* pronuclear injection (PNI) was published, announcing the first procedure for the production of transgenic farm animals (Hammer et al., 1985). About 10 years later, in 1996, success of cloning sheep from more differentiated embryonic cells has been reported (Campbell et al., 1996). One year later, the same group announced unprecedented success by cloning the sheep *Dolly* from adult somatic cells (Wilmut et al., 1997). In the same year, another remarkable advance had been achieved by using transfected fetal fibroblast cells for the generation of the first transgenic cloned sheep (Schnieke et al., 1997). Based on these advances, somatic cell nuclear transfer (SCNT) has been established as an essential tool for the creation of transgenic animals. Using these two approaches (PNI and SCNT), a large number of transgenic sheep and goats have been made for various purposes (**Tables 1** and **2**). From that time, various strategies have been applied to facilitate the generation of gene-modified animals that express specific and desired traits, employing spermatozoa, viral vectors, transposons, recombinases, RNA interference (RNAi) molecules, and endonucleases (**Figure 2**). Of these gene manipulation tools, the clustered regularly interspaced short palindromic repeat/CRISPR-associated protein 9 (CRISPR/Cas9) is currently revolutionizing the field of genome editing throughout virtually all biological kingdoms (Doudna and Charpentier, 2014; Hsu et al., 2014).

**Table 1:** Examples of transgenic sheep and goats produced using a pronuclear microinjection (PNI) approach.

Species	Construct abbreviation	Construct name	Main trait	References
Sheep	*mMT/hGH*	*Mouse metallothionein-I/human growth hormone*	Growth	Hammer et al., 1985
	*oMT/oGH*	*Ovine metallothionein-I/ovine growth hormone*	Growth	Murray et al., 1989
	*mMT/bGH*	*Mouse metallothionein-I/bovine growth hormone*	Growth	Rexroad et al., 1989
	*mMT/hGRF*	*Mouse metallothionein-I/human growth hormone–releasing factor*	Growth	Rexroad et al., 1989
	*mTRF/bGH*	*Mouse transferrin/bovine growth hormone*	Growth	Rexroad Jr. et al., 1991
	*mALB/hGRF*	*Mouse albumin/human growth hormone–releasing factor*	Growth	Rexroad Jr. et al., 1991
	*oBLG/hFIX*	*Ovine β-lactoglobulin/human factor IX*	Therapeutic proteins in milk	Clark et al., 1989
	*oBLG/hα1AT*	*Ovine β-lactoglobulin/human α1-antitrypsin*	Therapeutic proteins in milk	Wright et al., 1991
	*oBLG/hFVIII*	*Ovine β-lactoglobulin/human factor VIII*	Therapeutic proteins in milk	Niemann et al., 1996
	*vLRT/vvENV*	*Virus long terminal repeat/visna virus envelope*	Disease model	Clements et al., 1994
	*hHTT/hHTT*	*Human huntingtin/human huntingtin*	Disease model	Jacobsen et al., 2010
	*CMVp/oTLR4*	*Cytomegalovirus promoter/ovine toll-like receptor 4*	Disease resistance	Deng et al., 2012a
	*mKER/oIGF1*	*Mouse ultrahigh-sulfur keratin/ovine insulin-like growth factor 1*	Wool	Damak et al., 1996b
	*mKER/baCAT*	*Mouse ultrahigh-sulfur keratin/bacterial chloramphenicol acetyl transferase*	Wool	Damak et al., 1996a
Goat	*mWAP/hLAtPA*	*Mouse whey acidic protein/human longer-acting tissue plasminogen activator*	Therapeutic proteins in milk	Ebert et al., 1991
	*bβCas/hFIX*	*Bovine β-casein/human factor IX*	Therapeutic proteins in milk	Huang et al., 1998
	*cβCas/hAT*	*Caprine β-casein/human antithrombin*	Therapeutic proteins in milk	Edmunds et al., 1998
	*cβCas/hG-CSF*	*Caprine β-casein/human granulocyte colony-stimulating factor*	Therapeutic proteins in milk	Ko et al., 2000
	*cβCas/hLF*	*Caprine β-casein/human lactoferrin*	Therapeutic proteins in milk	Zhang et al., 2008a
	*bαS1Cas/hLZ*	*Bovine αs1-casein/human lysozyme*	Therapeutic proteins in milk	Maga et al., 2003
	*cβCas/hBChE*	*Caprine β-casein/human butyrylcholinesterase*	Therapeutic proteins in milk	Huang et al., 2007
	*bBLG/rSCD*	*Bovine β-lactoglobulin/rat stearoyl-coa desaturase*	Alteration of milk composition	Reh et al., 2004
	*CMVp/cTLR2*	*Cytomegalovirus promoter/caprine toll-like receptor 2*	Disease resistance	Deng et al., 2012b

**Table 2:** Examples of transgenic and gene-targeted sheep and goats produced using a somatic cell nuclear transfer (SCNT) approach.

Species	Gene or construct abbreviation	Gene or construct name	Main trait	References
Sheep	*oBLG/hFIX*	*Ovine β-lactoglobulin/human factor IX*	Therapeutic proteins in milk	Schnieke et al., 1997
	*oBLG/hα1AT*	*Ovine β-lactoglobulin/human α1-antitrypsin*	Therapeutic proteins in milk	McCreath et al., 2000
	*CAGp/ceFat1*	*Chicken β-actin promoter/Caenorhabditis elegans fat-1*	Enrich n-3 fatty acids	Duan et al., 2012
	*CMVp/oTLR4*	*Cytomegalovirus promoter/ovine toll-Like receptor 4*	Disease resistance	Deng et al., 2013
	oPrP*	*Ovine prion protein*	Disease resistance	Denning et al., 2001
Goat	*cβCas/hAT*	*Caprine β-casein/human antithrombin*	Therapeutic proteins in milk	Baguisi et al., 1999
	*bαS1Cas/hLF*	*Bovine αS1-casein/human lactoferrin*	Therapeutic proteins in milk	An et al., 2012
	*cβCas/hLF*	*Caprine β-casein/human lactoferrin*	Therapeutic proteins in milk	Wan et al., 2012
	*bβCas/hLZ*	*Bovine β-casein/human lysozyme*	Therapeutic proteins in milk	Liu et al., 2013c
	*cβCas/hLZ*	*Caprine β-casein/human lysozyme*	Therapeutic proteins in milk	Yu et al., 2013a
	*bBLG/hLZ*	*Bovine β-lactoglobulin/human lysozyme*	Therapeutic proteins in milk	Yu et al., 2013a
	*cβCas/hAFP*	*Caprine β-casein/human α-fetoprotein*	Therapeutic proteins in milk	Parker et al., 2004
	*cBLG/hPA*	*Caprine β-lactoglobulin/human plasminogen activator*	Therapeutic proteins in milk	He et al., 2018a
	*cβCas/hCuZn-SOD*	*Caprine β-casein/human copper-zinc superoxide dismutase*	Therapeutic proteins in milk	Lu et al., 2018
	*cβCas/hEC-SOD*	*Caprine β-casein/human extracellular superoxide dismutase*	Therapeutic proteins in milk	Lu et al., 2018
	*cBLG/hLA*	*Caprine β-lactoglobulin/human α-lactalbumin*	Valuable molecule in milk	Yuan et al., 2014
	*cβCas/pfMSP142*	*Caprine β-casein/Plasmodium falciparum merozoite surface protein 1*	Candidate malaria vaccine	Behboodi et al., 2005
	*cBLG/cGH*	*Caprine β-lactoglobulin/caprine growth hormone*	Improve milk production	Zhang et al., 2014b
	*cβCas/cIGF-1*	*Caprine β-casein/caprine insulin-like growth factor I*	Improve milk production	Lin et al., 2014
	*hEF1a1/eGFP*	*Human elongation factor-1α/enhanced green fluorescent protein*	Marker gene	Keefer et al., 2001
	*CMVp/DsRed*	*Cytomegalovirus promoter/Discosoma sp. red fluorescent protein*	Marker gene	Nuo et al., 2016
	*hsvTK/Neor*	*Herpes simplex virus thymidine kinase/neomycin*	Marker gene	Zou et al., 2002
	*mαMHC/hTGF-β1*	*Mouse alpha myosin heavy chain/human transforming growth factor-β1*	Disease model	Polejaeva et al., 2016
	*bβCas/hBD3*	*Bovine β-casein/human β-defensin-3*	Disease resistance	Liu et al., 2013b
	*CMVp/oAANAT*	*Cytomegalovirus promoter/ovine aralkylamine N-acetyltransferase*	Disease resistance	Tao et al., 2018
	cPRNP*	*Caprine prion protein*	Disease resistance	Yu et al., 2006
	cBLG*	*Caprine β-lactoglobulin*	Alteration of milk composition	Zhu et al., 2016a

*Gene targeting by homologous recombination.

CRISPR/Cas9 is a particularly simple, precise, and efficient gene-editing tool, which enabled its rapid and widespread application to different research fields. In our laboratory, CRISPR/Cas9 has been utilized to generate gene-edited sheep and goats both to investigate gene functions and to enhance economically important traits, such as muscle mass, fiber length, coat color, fat color, and litter size, among other traits (**Table 3**). In addition, a growing list of publications report applications of CRISPR/Cas9 in sheep and goats for other purposes such as manipulating the milk components, modeling human diseases, generating disease-resistant individuals, and developing hosts for growing human organs (**Figure 1** and **Table 3**). In general, CRISPR/Cas9 has been used in sheep and goats to introduce different forms of modifications including gene knockout, multiplex gene knockout, gene knockin, point mutation using DNA oligo templates, single-nucleotide alteration using base editors, simultaneous gene knockout and gene knockin, and deletion *via* dual single-guide RNAs (sgRNAs) (**Table 3**). In addition to the other toolkits of genome engineering, CRISPR systems have shown unprecedented potential for the generation of gene-edited animals with defined genetic alterations.

**Table 3:** Examples of gene-edited sheep and goats produced using the CRISPR/Cas9 system

Species	Gene(s)*	Editing type	Delivery method	Transferred embryos/recipients/pregnancies	Pregnancy rate**	Obtained founders^+^ (birth rate***)	Mutated founders^+^ (targeting efficiency****)	References
Sheep	*MSTN*	KO	MI	213/55/31	56.3%	35 (16.4%)	2 (5.7%)	Han et al., 2014
	*MSTN*	KO	MI	53/29/19	65.5%	22 (41.5%)	10 (45.4%)	Crispo et al., 2015a
	*MSTN, ASIP, BCO2*	M-KO	MI	578/82/34	41.4%	49 (8.4%)	36^++^ (73.4%)	Wang et al., 2016b^#^
	*MSTN*	KO	MI	130/N.A./N.A.	N.A.	32 (24.6%)	5 (15.6%)	Wu et al., 2018
	*MSTN*	KO	SCNT	415/20/8	40.0%	6 (1.4%)	3 (50.0%)	Zhang et al., 2018c
	*FGF5*	KO	MI	100/53/14	26.4%	18 (18.0%)	3 (16.6%)	Hu et al., 2017
	*FGF5*	KO	MI	170/101/20	19.8%	20 (11.7%)	16 (80.0%)	Li et al., 2017c
	*ASIP*	KO	MI	92/60/6	10.0%	6 (6.5%)	5 (83.3%)	Zhang et al., 2017a
	*BMPR1B (FecB^B^)*	PM	MI	279/39/16	41.0%	21 (7.5%)	7^+++^ (33.3%)	Zhou et al., 2018^#^
	*AANAT, ASMT*	KI	MI	593/150/77	51.3%	98 (16.5%)	34 (34.6%)	Ma et al., 2017
	*AANAT*	KI	MI	977/181/59	31.5%	79 (8.0%)	50 (63.2%)	Tian et al., 2018b
	*tGFP*	KI	MI	30/N.A./N.A.	N.A.	8 (26.0%)	1 (12.5%)	Wu et al., 2016
	*CFTR*	KO	SCNT	1029/73/34	46.5%	33 (3.2%)	33 (100.0%)	Fan et al., 2018
	*ALPL*	PM	MI	41/17/9	52.9%	15 (36.5%)	6^+++^ (40.0%)	Williams et al., 2018
	*SOCS2*	BE	MI	20/8/3	37.5%	4 (20.0%)	3^+++^ (75.0%)	Zhou et al., 2019#
Goat	*MSTN*	KO	SCNT	269/21/7	33.3%	3 (1.1%)	3 (100.0%)	Ni et al., 2014
	*MSTN, FGF5*	M-KO	MI	416/137/64	46.7%	93 (22.3%)	26^++^ (27.9%)	Wang et al., 2015^#^
	*MSTN*	KO	MI	18/5/3	60.0%	4 (22.2%)	1 (25.0%)	Guo et al., 2016
	*MSTN*	KO	MI	N.A./7/6	85.7%	8 (N.A).	6 (75.0%)	He et al., 2018b
	*fat-1, MSTN*	KI, KO	SCNT	134/56/8	14.2%	1 (0.74%)	1 (100.0%)	Zhang et al., 2018a
	*BLG*	KO	MI	103/67/18	26.8%	26 (25.2%)	4 (15.3%)	Zhou et al., 2017
	*GDF9*	PM	MI	56/17/13	76.4%	18 (32.1%)	6^+++^ (33.3%)	Niu et al., 2018^#^
	*EDAR*	KO	SCNT	257/79/5	6.3%	6 (2.3%)	6 (100.0%)	Hao et al., 2018
	*FGF5*	BE	MI	22/7/3	42.8%	5 (22.7%)	5^+++^ (100.0%)	Li et al., 2018b^#^

N.A., not available; KO, knockout; M-KO, multiplex knockout; KI, knockin; PM, point mutation (using HDR); BE, base editing; MI, microinjection; SCNT, somatic cell nuclear transfer; *(MSTN, myostatin also known as GDF8 growth differentiation factor 8; ASIP, agouti-signaling protein; BCO2, β-carotene oxygenase 2; FGF5, fibroblast growth factor 5; BMPR-1B “FecB”, bone morphogenetic protein receptor type 1B “Booroola fecundity gene FecB^B^ mutation”; AANAT, arylalkylamine N-acetyltransferase or SANT serotonin N-acetyltransferase; ASMT, acetylserotonin methyltransferase; tGFP, turbo green fluorescent protein; CFTR, cystic fibrosis transmembrane conductance regulator; ALPL, alkaline phosphatase biomineralization associated; SOCS2, suppressor of cytokine signaling 2; BLG, β-lactoglobulin; GDF9, growth differentiation factor 9; EDAR, ectodysplasin receptor); *indicates to the full names (in the table legend) of the used gene abbreviations in this column. **pregnancy rate (%) = no. of pregnancies/no. of recipients; ***birth rate (%) = no. of obtained founders/no. of transferred embryos; ****targeting efficiency (%) = no. of mutated founders/no. of obtained founders; ^+^alive and dead; ^++^total number of the obtained mutated founders whether they carry mutations in a single gene or in more than one gene; ^+++^the founders might be mutated but not all of them only show the defined point mutation. ^#^Models generated by our research team.

Menchaca et al. (2016) previously reported the tools used for the genetic modification of small ruminants. Here, we further extend this effort by presenting key examples of generated sheep and goat models and by providing an update on the evolution and potential of CRISPR/Cas9 applications in sheep and goats.

## Overview of Genetic Modification and Relevant Biotechnological Advances in Sheep and Goats

Tremendous advances in the field of genetic engineering in animals have been achieved over the past few decades. Various strategies have been used to generate genetically modified animals with desired traits (**Figures 1** and **2**). Increasing the efficiencies of mediating specific genetic modifications and simplifying the procedures for generating genetically modified organisms were the main aims that challenged specialists in this field. Enormous and collective efforts have been made in science and technology to facilitate the ability to induce specific genomic manipulations using procedures that have become more efficient and simpler to use. In the following paragraphs, the various tools for mediating genetic manipulations in sheep and goats (**Figure 2**) as well as some other relevant biotechnological advances are presented.

### The First Waves

#### Cloning by Embryo Splitting

Cloning by embryo splitting, also known as twinning by separation of blastomeres, is a set of primary reproductive techniques that have been applied to fulfill purposes related to the production of identical twins, multiplets, and chimeras. Embryo-splitting techniques were among the first strategies that led to the emergence of cloning. Based on the concept of cloning, researchers could produce genetically modified sheep and goats by using SCNT. Therefore, these multiplication strategies are discussed in this review. Previous work on sea urchins, salamanders, rats, rabbits, and mice have led to the application of these techniques in sheep and goats (Seidel, 1983; McLaren, 2000; Vajta and Gjerris, 2006). Sheep were among the first domestic animals that are subjected to embryo-splitting techniques. In 1979, Willadsen introduced a simple and successful procedure for blastomere separation in sheep (Willadsen, 1979). Willadsen aimed to develop new and highly selective methods for the breeding of farm animals, in addition to utilizing cells of cleaved embryos to increase the number of available embryos from superior mothers for the production of valuable offspring. Basically, the established procedure included the collection of cleaved embryos from super-ovulated donor females, blastomere separation, placement of each half into the zona pellucida, embedding in agar, and finally, transfer to recipient mothers. Willadsen summarized the results of ovine blastomere separation as follows: half embryos [single cells from two-cell embryos (1/2), pairs of cells from four-cell embryos (2/4), and a group of four cells from eight-cell embryos (4/8)] obtained ∼66% pregnancy rates. Quarter embryos [single cells from four-cell embryos (1/4), and pairs of cells from eight-cell embryos (2/8)] obtained ∼50% pregnancy rates. Eighth embryos (single cells from eight-cell embryos 1/8) obtained ≳5% pregnancy rates (Willadsen, 1989).

Embryo splitting was also applied to other livestock species including goats for both experimental and commercial purposes (Tsunoda et al., 1985; Udy, 1987). Different strategies have been adapted to improve the efficiency to produce an increased number of monozygotic animals. These strategies include the evaluation of isolated cells from different stages of development (Willadsen, 1980; Willadsen, 1981), presence or absence of intermediate hosts (Gatica et al., 1984), and presence or absence of zona pellucida (Shelton and Szell, 1988). The production of monozygotic animals *via* embryo splitting was a useful procedure for research and study of embryo development; however, the application of this procedure remained limited. Moreover, technical difficulties and suboptimal pregnancy rates lead to the production of only a relatively small number of individuals using this procedure. This is due to the limited divisibility of embryos to obtain two or occasionally up to four genetically identical animals. Although blastomere separation is considered as one of the basic cloning approaches, more promising approaches (such as nuclear transplantation) have opened the way for the large-scale production of genetically identical individuals.

#### Sheep–Goat Interspecific Chimerism

Despite the family relationship between sheep and goats, hybridization between both genera is an extremely rare event. Natural hybridization between sheep and goats is in most cases accompanied by high mortality rates of hybrid fetuses during the second month of pregnancy, likely as a result of fetal/maternal immunological incompatibility (Dent et al., 1971). By using an embryo aggregation strategy, researchers were able to produce sheep–sheep intraspecific chimeras (Tucker et al., 1974; Fehilly et al., 1984a). These fundamental studies indicated the aggregation ability of isolated blastomeres from early cleavage stage embryos to produce chimeric blastocysts that can be transferred to foster mothers (recipients) for the production of intraspecific chimeras. Based on these experiences and with the aim to increase the understanding of reproductive incompatibilities between species, in addition to providing a successful approach for interspecific hybridization, researchers were able to generate sheep–goat chimeras by using two basic techniques: embryo aggregation (Meinecke-Tillmann and Meinecke, 1984; Fehilly et al., 1984b) and embryonic cell injection into host blastocysts (Polzin et al., 1987). These procedures were based on the combination of blastomeres of two species, surrounding blastomeres of two species with each other, or injecting cells of the inner cell mass of one species into the blastocyst cavity of different species.

Sheep–goat chimeras differ from sheep–goat hybrids, which can be obtained when a goat naturally mates with a sheep. The phenotypic characteristics of sheep–goat chimeras include regions of both sheep-like wool and goat-like hair. Due to the mosaic nature of goat and sheep tissues in the produced chimeras, chimeric characteristics cannot be transferred to the next generation. Fertile sheep–goat chimeras can either pass on sheep or goat characteristics to their progeny depending on whether the reproductive organs of the chimera formed from caprine or ovine origins (Amoah and Gelaye, 1997). Interspecific chimerism may offer experimental approaches for developmental biology to investigate cell linkages, embryonic development interactions, reproductive incompatibilities, and embryo transfer opportunities. Although these approaches have been used to remove the reproductive barriers between species, the expanded use of these techniques to create new hybrids remained limited.

#### PNI

PNI was among the first techniques that have been applied to generate transgenic animals. It was the dominant methodology for the generation of transgenic animals during the first decade of animal transgenesis studies. By introducing DNA constructs into the pronuclei of fertilized eggs and transferring the injected eggs to foster mothers, researchers were able to generate transgenic animals (**Figure 2**). After reporting the generation of transgenic mice using this technique (Gordon et al., 1980; Gordon and Ruddle, 1981), in 1985, Hammer et al. (1985) were the first to report the generation of transgenic livestock. Sheep were among the first reported transgenic domestic animals; however, although Hammer et al. were able to generate a transgenic lamb, carrying the *mouse metallothionein-I/human growth hormone (mMT/hGH)* transgene, it did not express the integrated gene. The authors speculated that the reasons behind the low efficiencies might be due to the concentration of injected DNA, the composition of the used buffer, the stage of the collected embryos, and other structural aspects of the chromosomes. Further attempts have been made to overcome these obstacles and to facilitate the generation of transgenic animals using this technique. A few years later, in 1991, Ebert et al. (1991) reported the first generation of transgenic goats carrying the *mouse whey acidic protein/human longer-acting tissue plasminogen activator (mWAP/hLAtPA)* transgene using the same technique.

In both sheep and goats, the reported efficiencies of conventional PNI of DNA constructs were ∼1% of the injected zygotes (Clark, 2002). Several challenges caused these low efficiencies such as the random integration and the variable copy number of integrated DNA constructs. Because of these factors, the expression of the transgene can be unpredictable. A further technical challenge related to the application of PNI in livestock is the visualization of pronuclei. This is obstructed by the presence of a large amount of lipid granules in livestock eggs, which results in a nontransparent cytoplasm, thus hampering the localization of pronuclei. The pronuclei of ovine eggs can be visualized using differential interference contrast (DIC) microscopy (Hammer et al., 1985). In other species such as goats, a centrifugation step (12,000 × g for 5 min) improves the visualization of pronuclei (Freitas et al., 2016). Despite the suboptimal efficiencies of the conventional PNI, a large number of transgenic sheep and goats have been generated. Prominent examples of generated transgenic sheep and goats using PNI are shown in **Table 1**. The contributions of classical PNI of DNA constructs equipped the global transgenic sheep and goat sector with novel and useful genetically modified models. Furthermore, new forms of oocyte/zygote microinjection have emerged that provide simpler strategies for the introduction of desirable manipulations to the genomes of sheep and goats.

#### Embryonic Cell Cloning

Cloning can happen naturally in a number of living organisms *via* asexual reproduction and can also be artificially introduced in mammals by using primary techniques such as embryo splitting (Vajta and Gjerris, 2006). Efforts in embryo manipulation research led to the development of technical tools that enabled the production of identical individuals as well as intra- and inter-specific chimeric individuals (Willadsen, 1979; Fehilly et al., 1984a; Fehilly et al., 1984b). During the second wave of the development of these enabling technical tools, new and more advanced techniques emerged. Nuclear transfer, or nuclear transplantation, as it was first called, was developed to overcome the limitations of embryo-splitting techniques such as the limitation of the number of individuals that can be produced from a single split embryo. Despite the first attempts of nuclear transplantation in non-mammalian animal species and laboratory mice (Meissner and Jaenisch, 2006), the first cloned mammal (sheep) from undifferentiated embryonic blastomeres was reported in 1986 by Willadsen (Willadsen, 1986). Willadsen used ovine 8 to 16-cell stage embryos as nuclear donors in combination with ovine enucleated metaphase II oocytes as recipient cytoplasts to produce live lambs. Willadsen aimed to define suitable conditions required for the large-scale cloning of domestic animals. Despite the scientific significance of Willadsen’s work, academic and public attention was attracted later when more technically challenging cells have been used to produce viable cloned offspring (Vajta and Gjerris, 2006).

About 10 years later, in 1996, Campbell et al. reported the production of cloned lambs using long-term cultured and more differentiated embryonic cells rather than early embryonic or primary cultured embryo-derived cells (Campbell et al., 1996). Campbell et al. offered more choices for nuclei sources and attempted to overcome the limitations of the use of embryonic blastomeres (e.g., the limited number of this type of cells and their uncertain ability of long-term culture). This was in response to the unsuitability of the utilization of this type of cells in genetic modification programs (Colman, 1999). 1 year later, the same group reported pioneering and unprecedented work of utilizing fetal and adult mammalian cells to produce viable offspring, which resulted in the generation of the first and most famous somatic cloned animal in the world, the sheep *Dolly* (Wilmut et al., 1997). In the same year, a further advance has been accomplished by the generation of the first transgenic cloned sheep carrying *human coagulation factor IX (hFIX)* gene from transfected fetal fibroblasts (Schnieke et al., 1997). This success of cloning approaches in sheep was followed by several attempts to clone various species, including goats. Cloned goats were first produced by early embryonic blastomeres (Yong et al., 1991; Yong and Yuqiang, 1998). Subsequently, fetal somatic cells were used for the generation of transgenic cloned goats carrying *human antithrombin III (hAT)* gene (Baguisi et al., 1999). The aims of the cloning of domestic animals have been altered due to rapid and significant advances in the field. In addition to utilizing this approach as a valuable tool in embryological studies and to achieve the multiplication of desired genetics, nuclear transfer has become one of the basic methods to generate genetically modified animals with useful and desired traits.

#### Somatic Cell Cloning

Somatic cell cloning or SCNT has emerged with the creation of *Dolly*, the sheep from a mammary gland cell of a 6-year-old ewe, taken by Wilmut and his colleagues in 1997 (Wilmut et al., 1997). In addition to the putative aims of animal cloning such as multiplying superior animals for the construction of highly productive flocks for agricultural purposes and the restoration of endangered or even extinct species, the application of SCNT in genetic modification programs of farm animals has attracted wide attention. The emergence of SCNT has removed the barriers that inhibited the implementation of gene targeting by homologous recombination (HR) in species that lack embryonic stem cells (ESCs) to generate authentic genetically modified individuals. Moreover, implementation of SCNT for the manipulation of animal genomes has overcome several of the drawbacks of previously emerged PNI such as the low level of transgene integration, the variability of transgene expression, the unpredictable transmission of the transgene to the next generation, and founder mosaicism.

Transgenic farm animals can be produced using SCNT *via* transfection of donor cell nuclei with DNA expression constructs or vectors or by cloning transgenic founder animals (**Figure 2**). Various cell types have been utilized as nucleic donors to generate cloned sheep and goats. These include adult mammary gland cells (Wilmut et al., 1997), adult granulosa cells (Keefer et al., 2002; Loi et al., 2002), adult cumulus cells (Zou et al., 2001), fetal fibroblast cells (Wilmut et al., 1997; Baguisi et al., 1999), and other potentially utilizable cells such as fetal skeletal muscle–derived satellite cells (Ren et al., 2014). Fetal fibroblast cells have been used dominantly for the generation of transgenic cloned sheep and goats among other reported cell types. After the first generation of transgenic cloned sheep reported in 1997 (Schnieke et al., 1997), and the first transgenic cloned goats in 1999 (Baguisi et al., 1999), a large number of transgenic and gene-targeted cloned sheep and goats have been generated. Examples of generated transgenic sheep and goats using SCNT are listed in **Table 2**. Despite the relatively low efficiency of SCNT and the potential for developmental anomalies, in parallel with the PNI technique, SCNT has become a basic and dominant methodology to generate transgenic and gene-targeted sheep and goats.

#### Interspecific Cloning

In addition to the promising advantages of SCNT for the multiplication of genetically valuable or superior livestock and the manipulation of the genomes of experimentally, biomedically, and agriculturally important animals, SCNT offers promising potential for the conservation of genomes of endangered species and for restoring or reviving the genomes of extinct species. Finding effective tools to conserve and restore threatened genomes is equally important to finding new tools for the manipulation of existing genomes to generate novel and desirable phenotypes. Interspecies cloning or interspecies somatic cell nuclear transfer (iSCNT) is one of the emerging strategies to conserve genetic diversity and prevent the rapid loss of animal genetic resources. Genetic rescue programs based on iSCNT use nuclei from endangered species in the wild, whose oocytes are difficult to obtain, with oocytes from closely related domesticated species to reconstruct embryos that can then be transferred to foster mothers. The resultant offspring of this process resembles nucleic donors.

Sheep and goats were among the closely related domesticated species that were utilized in the conservation cloning programs of threatened species that belong to the genera *Ovis and Capra*. Loi et al. (2001) reported the successful generation of a cloned mouflon from reconstructed embryos, combining mouflon *post mortem* somatic cells, and domestic sheep oocytes. This provided an encouraging example of the application of iSCNT for the generation of live founders. Further examples of the implementation of iSCNT using sheep and goats to reconstruct embryos between species within the same genera are listed in **Table 4**. Despite the successful attempts of reconstructing embryos between two species from closely related genera, the number of viable offspring produced using this strategy was very low. Such low efficiency might be a result of implantation failure or of immunological rejection (Wang et al., 2001). Other embryonic combinations have also been reported for the study of developmental ability, mitochondrial heteroplasmy, and nuclear-cytoplasmic interactions between different species (**Table 4**). Despite the low numbers of publications reporting the successful generation of viable offspring using iSCNT, research in this field still offers great potential in interspecies embryological studies and genetic resource conservation programs.

**Table 4:** Examples of interspecies somatic cell nuclear transfer (iSCNT) applications in sheep and goats for the reconstruction of embryos between different species.

Intra-/Inter-genera	Nucleus donor	×	Oocyte donor	References
Within the same genus	Argali (*Ovis ammon*)	×	Sheep (*Ovis aries*)	White et al., 1999
	European mouflon (*Ovis orientalis musimon*)	×	Sheep (*Ovis aries*)	Loi et al., 2001
	Esfahan mouflon (*Ovis orientalis isphahanica*)	×	Sheep (*Ovis aries*)	Hajian et al., 2011
	Ibex (*Capra ibex*)	×	Goat (*Capra hircus*)	Wang et al., 2007
Between different genera	Tibetan antelope (*Pantholops hodgsonii*)	×	Goat (*Capra hircus*)	Zhao et al., 2007
	Goat (*Capra hircus*)	×	Sheep (*Ovis aries*)	Ma et al., 2008
	Goat (*Capra hircus*)	×	Bovine (*Bos taurus*)	Tao et al., 2008
	Goat (*Capra hircus*)	×	Buffalo (*Bubalus bubalis*)	Selokar et al., 2011
	Sheep (*Ovis aries*)	×	Bovine (*Bos taurus*)	Hua et al., 2008
	Human (*Homo sapiens*)	×	Goat (*Capra hircus*)	Sha et al., 2009
	Human (*Homo sapiens*)	×	Sheep (*Ovis aries*)	Hosseini et al., 2012

#### Handmade Cloning

Handmade cloning (HMC) is a simplified version of the SCNT technique. This modified technique has emerged to overcome the technical difficulties that obstruct the improvement and widespread application of SCNT. In traditional SCNT, oocyte enucleation is one of the technical steps which encounter major obstacles due to the presence of the zona pellucida (the outer thick membrane of mammalian oocytes) (Lagutina et al., 2007). Thus, introducing careful manipulations inside the zona pellucida to replace the nuclei (enucleating the oocyte nucleus and transferring the somatic cell nucleus) requires expensive instruments such as micromanipulators as well as both skill and time (Vajta and Gjerris, 2006). The simplicity of HMC is mainly based on the removal of the zona pellucida after maturation and before enucleation. In this case, sophisticated micromanipulators are not necessary because the manipulations required for both enucleation and nucleus transfer are performed by hand as indicated by the name (Vajta, 2007). Basically, the procedure of HMC includes handmade bisection of zona-free oocytes, staining and selection of cytoplasts, and fusion of the somatic cell with two cytoplasts to generate an equally sized reconstructed embryo (Vajta et al., 2006). Thus, the implementation of SCNT using this approach requires less expertise, time, and cost.

Initial attempts to use zone-free procedures, especially in embryonic cell nuclear transfer, have led to the first successful report to produce cloned cattle, using a somatic cell as nucleus donor (Vajta et al., 2001). In sheep and goats, initial publications have reported the application of this technique for successful embryo development (Peura and Vajta, 2003; Akshey et al., 2008), followed by a number of publications reporting the application of this technique to produce cloned (Malik et al., 2014; Khan et al., 2018), transgenically cloned (Lagutina et al., 2007; Pereira et al., 2013), or interspecies cloned embryos (Selokar et al., 2011; Yu et al., 2011). An interesting example of the application of this technique in sheep is the generation of transgenic cloned lambs carrying a *modified nematode mfat-1* gene to enrich muscles and other organs and tissues with omega-3 fatty acids (Zhang et al., 2013). In this study, one of the three generated founders showed a lower omega-6/omega-3 ratio, indicating the converting role of the integrated *mfat1 gene*. Despite the relatively equivalent efficiency of HMC compared to traditional SCNT, as well as the further advantages of HMC (simpler to use, cheaper, and more time-saving), the applications of HMC in sheep and goats to produce viable founders have not been studied in detail. In general, despite the simplification provided by HMC and other emerging strategies, SCNT remains technically challenging, and few research groups around the world are able to perform it efficiently (Tan et al., 2016). Further advances in this field are required to enable the widespread application of these techniques to facilitate multiplication, transgenesis, and genetic rescue of threatened genomes.

### Additional Tools for Transfer and Manipulation

#### Sperm-Based Transgenesis

Spermatozoa have the natural ability to obtain exogenous DNA by a simple incubation procedure (Brackett et al., 1971; Lavitrano et al., 1989). This significant observation opened the way for further alternative strategies that can be utilized in transgenesis programs. Basically, three main strategies have been used to mediate transgenesis that utilize the male side (spermatozoon), namely, male germ cell transplantation–mediated transgenesis, sperm-mediated gene transfer (SMGT), and testis-mediated gene transfer (TMGT) (**Figure 2**).

In addition to its importance for spermatogenesis and fertility studies, male germ cell transplantation has been suggested to be an alternative tool to mediate transgenesis. Spermatogonial transplantation uses isolated spermatogonial stem cells (SSCs) from desirable male donors and injects and transplants these into the seminiferous tubules of an infertile recipient males, which results in donor-derived spermatogenesis. In this case, transgenesis can be mediated by manipulating SSCs prior to transplantation into the recipient males or by transferring transgenic donor germ cells from transgenic individuals (Hill and Dobrinski, 2006). This technique has been initially established in rodents (Brinster and Avarbock, 1994; Brinster and Zimmermann, 1994). Although this technique has basically been extended to farm animals including goats (Honaramooz et al., 2003a; Honaramooz et al., 2003b; Zeng et al., 2012) and sheep (Rodriguez-Sosa et al., 2006), the application of this technique to produce transgenic founders is limited. A recent study in a pig model used genetic manipulation *via* CRISPR/Cas9 for the generation of male recipient models for SSC transplantation by targeting the nanos *C2HC-type zinc finger 2* (NANOS2) gene (Park et al., 2017). Homozygous knockout males showed an ablation of the male specific germline with intact testicular development, while heterologous knockout males and females were fertile. This offers an advantage in agriculture where these models serve as recipients for donor spermatogonial stem cells from genetically valuable males, thus expanding the availability of desirable genetics.

SMGT is directly based on the intrinsic ability of sperm cells to capture and internalize exogenous DNA (Lavitrano et al., 2006). After a simple step of incubating sperm cells with exogenous DNA, transfected sperm cells can then be transferred to female (eggs) using various strategies, such as artificial insemination (AI) (Zhao et al., 2010), intracytoplasmic sperm injection (ICSI), *in vitro* fertilization (IVF), or laparoscopic insemination (LI) (Pereyra-Bonnet et al., 2011), with varied efficiencies. SMGT application in sheep and goats includes the production of both transgenic embryos (Pereyra-Bonnet et al., 2008; Shadanloo et al., 2010; Pereyra-Bonnet et al., 2011; Pramod et al., 2016) and transgenic founders (Zhao et al., 2010) using marker transgenes. Despite the simplicity this approach offers, its application for the generation of transgenic sheep and goats remained limited. This might be a result of a number of drawbacks of this approach such as the low incorporation of the exogenous genes. In general, several attempts have been reported to enhance the ability of sperm to obtain exogenous DNA. These include electroporation-, liker-, retroviral-, liposome (lipofection)-based SMGT, restriction enzyme–mediated integration, and further techniques (Smith, 2012). The optimization of this approach might increase its efficiency.

TMGT or intratesticular injection are further alternative tools based on the direct injection of testes with exogenous DNA. After a specific interval, injected males can then be used to naturally mate with females to produce transgenic founders. TMGT was initially applied in sheep and goats to produce transgenic founders with inserted genes including *lipoprotein lipase (LPL)* (Qin et al., 2012), *solute carrier family 7 member 11 (SLC7A11)* (He et al., 2012), *peroxisome proliferator–activated receptor gamma (PPARγ)* (Qin et al., 2013), *myogenin (MyoG)* (Zhang et al., 2014c), and *enhanced green fluorescent protein (eGFP)* (Raina et al., 2015; Pramod and Mitra, 2018). In general, despite the potential possibilities of sperm-based strategies to mediate transgenesis, these strategies still require optimization. Integration between these strategies and newly emerging targeting tools might be of great importance for the generation of desirable genetically modified sheep and goats.

#### Virus-Based Transgenesis

Viral vectors have been used to mediate transgenesis by delivering and integrating transgenes into the host genome. Viral vectors can be divided into non-integrating viral vectors (e.g., adenoviral vectors), and integrating viral vectors that are mostly derived from a retrovirus, lentivirus, and adeno-associated virus (AAV) (Pfeifer and Hofmann, 2009). Viral vectors have been used after being made replication-deficient by deleting genes that are essential for viral pathogenesis and/or replication (Nakagawa and Hoogenraad, 2011).

Serval strategies have been utilized that use of viral vectors as vehicles or carriers to deliver chosen exogenous DNA constructs into targeted expression positions (**Figure 2**). One of the basic strategies that uses viral vectors is the direct intramammary injection *via* the teat canal for the transient production of valuable proteins in milk. Goats are an ideal model for the production of pharmaceutical molecules in milk and have been subjected to this protocol. *Human growth hormone (hGH)* was among the first published genes to be infused into the goat mammary gland using retroviral vectors (Archer et al., 1994). The same strategy has also been applied using adenoviral vectors to direct the expression of functional proteins into mammary secretory epithelial cells. Examples of the adenoviral vector–mediated transfer of genes infused *via* the teat canal of goat mammary glands are listed in **Table 5**.

**Table 5:** Examples of adenoviral-mediated gene transfer into the teat canal of caprine mammary glands.

Gene abbreviation	Gene full name	References
*Lys*	*Lysostaphin*	Fan et al., 2002
*hGH*	*Human growth hormone*	Sánchez et al., 2004; Han et al., 2009
*hEPO*	*Human erythropoietin*	Toledo et al., 2006; Liu et al., 2010
*hLTF*	*Human lactoferrin*	Han et al., 2007
*CSFV-E2*	*Classical swine fever virus E2*	Toledo et al., 2008; Sánchez et al., 2014
*hNGF-β*	*Human nerve growth factor beta*	Xiao et al., 2009
*hAT*	*Human antithrombin*	Yang et al., 2009
*hGCase*	*Human glucocerebrosidase*	Tavares et al., 2016

A further strategy that involves the transduction of goat male germline stem cells with an adeno-associated viral vector carrying *eGFP* marker gene, resulted in transgene transmission after germ cell transplantation (Honaramooz et al., 2008). Lentiviral vectors carrying the *eGFP* marker gene have also been applied to transduce sheep (Rodriguez-Sosa et al., 2009) and goat (Abbasi et al., 2015) spermatogonia prior to transplantation and colonization into male recipients. This approach might be a useful tool for the generation of transgenic founders, in particular, since it requires minimal embryo handling.

Viral vectors coupled with an RNAi mechanism have been used to mediate the loss of gene expression for the investigation of potential biological functions of genes, to suppress the expression of disease-related genes, and to inhibit the expression of genes that negatively regulate economically important traits. Recombinant adenoviruses that carry short hairpin RNA (shRNA) and that target goat *parathyroid hormone–related protein (PTHrP)* in mammary epithelial cells successfully inhibited *PTHrP* gene expression (Zheng et al., 2013). Lentiviral vector–based delivery of shRNA has also been used in both ovine and caprine cells to suppress the expression of target genes (**Table 6**). Additionally, injecting AAV9-miRNA targeting *human huntingtin (HTT)* in the striatum of transgenic Huntington’s disease (HD) sheep so that these express the full-length HTT gene reduced the mRNA and protein of human HTT by 50–80% in the striatum at 1 and 6 months postinjection (Pfister et al., 2018).

**Table 6:** Examples of ovine and caprine targeted gene expressions using RNA interference (RNAi).

Species	Gene abbreviation	RNAi molecule	Delivery tool	Cell type	References
Sheep	*MSTN*	shRNA	Lentiviral vector	Myoblasts	Liu et al., 2012; Liu et al., 2014
	*MSTN*	shRNA	Lentiviral vector	Fibroblasts	Tang et al., 2012
	*MSTN*	siRNA	Synthesized construct	Fibroblasts	Lu et al., 2012
	*TRIM28*	siRNA	Synthesized construct	Fibroblasts	Luo et al., 2017
	*INHα*	siRNA	Synthesized construct	Granulosa cells	Li et al., 2017a
Goat	*MSTN*	shRNA	Lentiviral vector	Fibroblasts	Lu et al., 2013; Patel et al., 2015
	*MSTN*	shRNA	Lentiviral vector	Myoblasts	Patel et al., 2014; Kumar et al., 2018
	*MSTN*	shRNA	Expression construct	Fibroblasts	Jain et al., 2010; Singh et al., 2012; Jain et al., 2015; Hati Boruah et al., 2016
	*MSTN*	shRNA	Expression construct	Myoblasts	Tripathi et al., 2013
	*MSTN*	siRNA	Synthesized construct	Myoblasts	Kumar et al., 2014
	*MSTN*	miRNA	Expression construct	Fibroblasts	Zhong et al., 2014
	*PrP*	shRNA	Lentiviral vector	Fibroblasts	Golding et al., 2006
	*BLG*	shRNA	Lentiviral vector	Fibroblasts	Zhang et al., 2012
	*PPARγ*	shRNA	Lentiviral vector	Fat cells	Du et al., 2018
	*PGC-1α*	shRNA	Lentiviral vector	Granulosa cells	Zhang et al., 2016a
	*DNMT1*	shRNA	Expression construct	Fibroblasts	Lan et al., 2010
	*FKBP38*	shRNA	Expression construct	Fibroblasts	Fu et al., 2015
	*BZW2*	shRNA	Expression construct	Mammary epithelial cells	Sun et al., 2012

shRNA, short hairpin RNA; siRNA, small interfering RNA; miRNA, microRNA; MSTN, myostatin; TRIM28, tripartite motif containing 28; INHα, inhibin α-subunit; PrP, prion protein; BLG, β-lactoglobulin; PPARγ, peroxisome proliferator-activated receptor γ; PGC-1α, peroxisome proliferator-activated receptor γ coactivator-1 α; DNMT1, DNA methyltransferase 1; FKBP38, FK506-binding protein 38; BZW2, basic leucine zipper and W2 domains 2.

Viral vectors can also be used to mediate transgenesis into zygotes by using two main ways: viral transduction of zona-free embryos and perivitelline space (subzonal) injection (Pfeifer and Hofmann, 2009). Both strategies have been applied using lentiviral vectors in sheep. Ritchie et al. have applied a lentiviral transduction protocol with *eGFP* marker gene for both zona-free and split embryos and reported potential possibilities for the application of this approach in small ruminants (Ritchie et al., 2005; Ritchie et al., 2009). Perivitelline space injection of lentiviral vectors that carry *eGFP* marker genes has shown efficient expression of green fluorescence in transgenic lambs (Liu et al., 2013a; Crispo et al., 2015b). Despite these results, using a 2A peptide–based tricistronic lentiviral vector for the expression of three fluorescent protein genes subjected to hypermethylation and silenced the expression of transgenes in transgenic sheep (Tian et al., 2013). Furthermore, cells derived from these transgenic sheep could achieve expression of transgenes when the epigenetic status has been regulated *via* methyltransferase and deacetylase inhibitors (Tian et al., 2013).

Other interesting examples include the production of transgenic sheep *via* perivitelline space injection of lentiviral vectors encoding shRNAs that have been specifically designed to inhibit the replication of foot-and-mouth disease virus (FMDV), or to silence the expression of *myostatin (MSTN)* to promote muscle growth (Cornetta et al., 2013). This report also provides additional safety information in support of the application of this technology. Additionally, recombinant lentivirus carrying the *fibroblast growth factor 5–short alternative transcript (FGF5s)* was used to produce transgenic sheep with enhanced fiber growth (Li et al., 2017b). Viral vector–based transgenic strategies have shown moderate efficiency compared to other conventional methodologies that have been used to mediate transgenesis. Despite the moderate efficiency of these strategies, newly emerging gene modifying tools are likely a better choice or are at least simpler and safer to use.

#### Recombinases

Recombinases are enzymes that promote site-specific genetic recombination. Recombinases are derived from nature and possess the ability to perform deletions, insertions, and inversions into DNA sequences *via* interaction between the recombinases and their own recognition sites (Olorunniji et al., 2016). Site-specific recombinases have been integrated into genome engineering programs for a variety of purposes. Manipulations using recombinases have been applied to sheep and goat genomes *via* Cre recombinase, Flp recombinase, and PhiC31 integrase (**Figure 2**).

In general, the Cre/*lox*P system uses bacteriophage P1-derived Cre recombinase, which acts on a 34-bp sequence called *lox*P. This 34-bp sequence consists of two 13-bp inverted or palindromic repeats separated by an 8-bp spacer region (Hoess and Abremski, 1984). The Cre/*lox*P site–specific recombination system was used to excise the selectable genes from goat transgenic cells (Xu et al., 2008). Xu et al. have combined both the Cre/*lox*P recombination system and protein transduction technology and produced TAT-Cre recombinase, which is a recombinant cell-permeable fusion protein. This recombinant protein was used to optimize the efficiency of delivery and to eliminate cytotoxic and genotoxic effects of both the integration and continuous expression of Cre recombinase-expressing vectors (Xu et al., 2008). Briefly, the authors used primary skin fibroblasts from *β-lactoglobulin (BLG)* transgenic goats that carried both the *human lysozyme (LYZ)* vector and selectable gene-expressing vector, which contained a left *lox*P site, neomycin resistance gene *Neo*, a thymidine kinase gene *TK*, and a right *lox*P site. After TAT-Cre protein transduction, one of the TAT-Cre treated cell colonies was used as a nuclear donor to perform nuclear transfer. Two selectable gene-free cloned goats were produced with removed *Neo/TK* cassette (Xu et al., 2008).

The yeast *Saccharomyces cerevisiae* derived Flp/*FRT* system functions in an analogous fashion to the Cre/*lox*P system, in which flippase (Flp) recognizes and cleaves two *FRT* recognition sites (Rafferty and Quinn, 2018). The Flp/*FRT* site–specific recombination system has been applied in goat somatic cells to mediate the site-specific integration and to eliminate the problematic random integration of transgenes (Yu et al., 2013b). Briefly, Yu et al. first performed gene targeting by HR thus introducing an *FRT*-docking site into the *α1 (I) procollagen (ColA1)* locus (HR efficiency of 5.9% “11/185”). Cell clones with successful targeting have been subjected to embryo cloning to achieve rejuvenation or regeneration. Cells with the *FRT*-homing site were isolated from cloned fetuses and co-transfected with two vectors, an *eGFP* replacement vector, and an Flp recombinase-expressing vector. After the second round of recombination, transgenic cells exhibited functional expression of *eGFP* with a gene replacement efficiency of 38.4% (15/39) (Yu et al., 2013b).

*Streptomyces* bacteriophage–derived ΦC31 (PhiC31) integrase has been applied to mediate HR between *attB* and corresponding pseudo *attP* sites (Andreas et al., 2002). Three pseudo-*attP* sites were identified in the sheep genome (Ni et al., 2012), while eight pseudo-*attP* sites were identified in the goat genome (Ma et al., 2014). These pseudo-*attP* sites were located in intron or intergenic regions. PhiC31 integrase has been applied in sheep (Ni et al., 2012) and goat (Ma et al., 2014) primary fibroblasts to integrate the *eGFP* marker cassette, showing an increase in gene integration efficiency. Recombinases have been used to perform modifications such as selectable cassette excision or the integration of marker or overexpression cassettes. Further strategies can also be used to achieve desirable modifications in a relatively simple and efficient manner.

#### Transposons

Transposable elements or transposons have been used for genetic manipulation after highlighting their role as active non-viral DNA delivery systems (Largaespada, 2003). These systems have been applied to sheep and goat genomes such as the *Sleeping Beauty* transposon and the *PiggyBac* transposon (**Figure 2**). The *Sleeping Beauty* transposon system works through the mediation of transposase to directly integrate *Sleeping Beauty* transposon into, mainly, thymine-adenine TA-dinucleotide sites of the target genome (Ivics et al., 1997). In sheep, the *Sleeping Beauty* transposon system was used in combination with RNAi to knockdown *MSTN* gene expression (Hu et al., 2011). *Sleeping Beauty*–mediated shRNA expression in transfected sheep fetal fibroblasts showed a significant decrease of *MSTN* expression, exceeding that of the random integration of anti-*MSTN* shRNA (Hu et al., 2011). This highlights the potential for the combination of the *Sleeping Beauty* transposon with RNAi to generate knockdown donor cells for animal cloning. This combined system was also used to generate transgenic sheep with resistance to FMDV by knocking down the FMDV-*VP1* gene (Deng et al., 2017). After pronuclear microinjection, eight out of 92 generated lambs showed positive integration of *VP1*-shRNA. This study also reported that shRNA mediated by the *Sleeping Beauty* transposon achieved an increased integration rate over random integration of anti-*VP1* shRNA. Furthermore, *Sleeping Beauty* transposase and Tn5 transposase were cytoplasmically injected into sheep zygotes to integrate transposon containing *recombinant human factor IX (rhFIX)* driven by the *BLG* promoter (Bevacqua et al., 2017). No transgenic lambs have been obtained from Tn5 transposase injection, while injection of *Sleeping Beauty* transposase resulted in two lambs that carried the transgene 2/7 (29%) (Bevacqua et al., 2017). More animals have to be produced for the accurate detection of Tn5 efficiency.

Another interesting transposon system, called the *PiggyBac*, has been applied by our team in cashmere goats. The *PiggyBac* transposon system was tested to mediate *eGFP* gene expression and to generate stably transfected cell lines with the use of goat fetal fibroblasts (Bai et al., 2012). The generated cell lines have shown a high level of *eGFP* mRNA. Furthermore, transfected cells with the same set of vectors (*eGFP*/*Neo* gene replacement vector and *PiggyBac* transposase–expressing vector) were used as donor cells to produce transgenic goats *via* SCNT (Bai et al., 2017). 14 cloned embryos were implanted into 20 recipient females; however, only one live transgenic kid was produced with a strong expression of the *eGFP* gene in the horns, hooves, nose, and hair. The *PiggyBac* transposon system has also been used to mediate overexpression of the *thymosin β-4 (Tβ4)* gene to improve the production of fine hair in cashmere goats (Shi et al., 2017). The hair follicle-specific *keratin-associated protein 6.1 (KAP6.1)* promoter was used to construct the *Tβ4*-overexpressing cassette. Using the same strategy, transfected goat fetal fibroblasts with the integration of *Tβ4* gene–expressing cassette have been used as donor cells to generate transgenic goats *via* SCNT (Shi et al., 2017). Five transgenic cloned founders were produced and showed an increased number of secondary hair follicles that produce the commercially desirable fine cashmere hair. Transposon systems have shown potential for the mediation of integrations that can result in gene knockdown or gene overexpression. In addition to the newly emerging toolkits of genetic engineering, transposons can contribute to the generation of desirable genetically modified cell lines and organisms.

#### RNAi

RNAi is an interesting natural mechanism that has been applied in genetic manipulation programs due to its ability to simply suppress (knockdown) gene expression by silencing the mRNA of the targeted gene. RNAi has mainly been used to understand the biological and functional roles of specific genes, investigate the inhibitory effects of gene expression, suppress the expression of pathogenic genes, and inhibit the expression of genes that negatively regulate desirable phenotypes. shRNA and small interfering RNA (siRNA) are the main forms of RNAi molecules that have been used to mediate gene knockdown in sheep and goat genomes. In sheep and goats, a set of strategies has been used to deliver RNAi-expressing constructs, to mediate the generation of knockdown cell lines, and to obtain transgenic individuals. These methods include the transfection of RNAi-expressing vectors, viral vectors (mainly lentiviral vectors), transposons (e.g., the *Sleeping Beauty* transposon system), SCNT, PNI, intratesticular injection, and direct injection into the targeted organ (**Figure 2**).

To equip sheep and goat models with potential disease resistance, RNAi has been used to suppress the expression of goat *prion protein (PrP)*. The examined brain tissues of a transgenic cloned fetus from lentiviral-shRNA transfected fibroblasts showed a significant decrease in *PrP* expression (Golding et al., 2006). Tongue epithelium cells isolated from transgenic goats carrying shRNA against the FMDV *3Dpol* gene showed eﬀective resistance after FMDV challenge (Li et al., 2015). Moreover, ear fibroblasts isolated from transgenic lambs that carried shRNA against the FMDV-*VP1* gene showed a significant inhibitory effect on the *VP1* gene (Deng et al., 2017). These studies highlight the possibilities for the use of RNAi strategies to confer potential disease resistance in farm animals.

In addition to providing models with resistance to epidemic diseases, the provision of models with enhanced economical traits is of great importance. Disrupting the normal function of the negative muscle-mass regulator *MSTN* offers promising potential for the promotion of meat production. Increasing animal muscle growth and body weight is one of the ultimate goals in agriculture. The *MSTN* gene is an attractive target to fulfill this purpose. Many studies have been published about the disruption of ovine and caprine *MSTN* using RNAi and a significant decrease of *MSTN* expression has been reported (**Table 6**). Moreover, *MSTN*-shRNA-expressing transgenic lambs have been produced *via* SCNT and showed a faster increase in body weight than control individuals (Hu et al., 2013).

**Figure 3:**
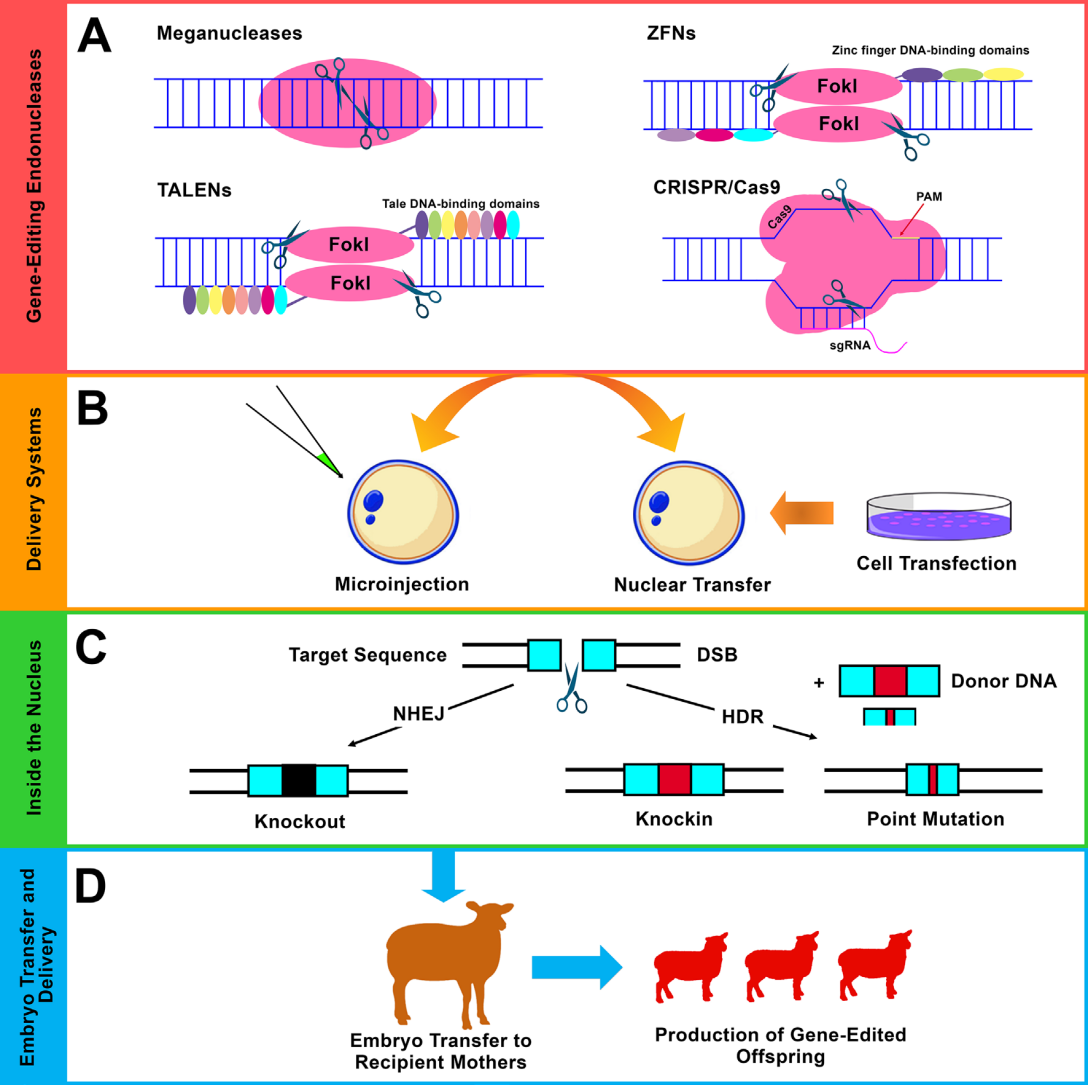
Gene editing using site-specific endonucleases. **(A)** The four major classes of endonucleases: meganucleases, ZFNs, TALENs, and CRISPR/Cas9. *Flavobacterium okeanokoites*, type IIS restriction enzyme (*Fok*I), protospacer adjacent motif (PAM), and single-guide RNA (sgRNA). **(B)** The commonly used delivery approaches of the endonucleases are the direct microinjection into embryos (mainly, cytoplasmic injection in sheep and goats) and somatic cell nuclear transfer (SCNT) (mainly, using fibroblast cells). **(C)** Different forms of modifications that result from the two main DNA repair pathways after induction of double-strand break (DSB) using endonucleases. i) Non-homologous end joining (NHEJ), which results in small insertions and/or deletions (indels), leads to gene knockout (disruption). ii) Homology-directed repair (HDR), which acts in the presence of exogenous donor DNA and mediates precise genetic modification including knockin (site-specific integration) and point mutation (single-nucleotide alteration). **(D)** Embryo transfer, gestation, and the generation of genetically edited offspring.

In addition, a set of genes have also been investigated using RNAi in a variety of ovine and caprine cells. Examples of these genes are shown in **Table 6**. A further strategy is based on the injection of testis with shRNA vectors, which was performed to target the *zinc finger protein Y-linked (ZFY)* gene. This strategy was applied as a genetic method of sex control and to bias the sex ratio toward females in sheep (Zhang et al., 2018b). In general, RNAi-based approaches are an interesting tool for mediating loss of gene expression by targeting the products of gene transcription (mRNA). Currently, more advanced strategies can be easily and directly applied to knockout the gene sequence instead of its mRNA, thus ensuring complete disruption of the associated gene function (**Figure 3**).

### The Recent Revolution

#### Meganucleases

Site-specific endonucleases are enzymes that can break down polynucleotide chains and make cleavages in DNA sequences. Generally, there are four main classes of endonucleases that have been utilized in gene-editing programs: i) meganucleases or homing endonucleases, ii) zinc finger nucleases (ZFNs), iii) transcription activator-like effector nucleases (TALENs), and (iv) CRISPR systems (**Figures 2** and **3A** ) (Hsu et al., 2014). Of these four different classes, three are commonly used: ZFNs, TALENs, and CRISPR systems. The use of endonucleases has attracted significant attention among scientists after the role of double-strand breaks (DSBs) in increasing the efficiency of HR event has been highlighted (Rouet et al., 1994; Choulika et al., 1995). Gene editing using endonucleases is based on their ability to promote DSBs. After such induction of DSBs, the DNA repair mechanisms function in different pathways depending on the situation and generates different forms of manipulations. In the absence of donor DNA, non-homologous end joining (NHEJ) functions *via* random formation of small insertions and/or deletions (indels) that result in gene disruption (knockout). In the presence of donor DNA, homology-directed repair (HDR) functions to mediate more precise modifications such as site-specific integration (knockin) and single-base alteration (point mutation) (**Figure 3C**) (Gaj et al., 2013). Other forms of modifications, such as large deletions and inversions, can also be achieved using optimized endonucleases. In addition to NHEJ and HDR, a single-strand annealing (SSA) repair mechanism can also be utilized to mediate modifications by inducing the deletion of an intervening fragment between two homogenous repeat sequences (Li et al., 2018c).

Meganucleases are rare-cutting enzymes that can be classified as the first class of sequence-specific nucleases. These have been employed to create targeted DSBs in eukaryotic genomes (Daboussi et al., 2015). Meganucleases include five families, and the family LAGLIDADG is the largest and best characterized. This family also contains the most specific cutters, such as *I-SceI* of yeast *S. cerevisiae* (Galetto et al., 2009). Strategies have been developed to engineer meganucleases with new properties and DNA-binding specificities to widen their applications (Galetto et al., 2009). Meganucleases have mostly been applied to experimental model organisms. However, the number of publications regarding the applications of meganucleases in farm animals is limited (Bevacqua et al., 2013; Wang et al., 2014), and no publications can be found regarding their application in sheep and goats. Presumably, challenges related to both the engineering of meganucleases and protein redesign to direct meganucleases to novel DNA sequences result in their limited application in farm animals including sheep and goats (Petersen, 2017). In addition to the initial role of meganucleases for understanding the significance of DSB-based gene-editing events, simpler and more customizable gene-editing tools such as ZFNs, TALENs, and CRISPR systems have emerged, providing further options and opportunities to apply gene editing to a wide range of organisms.

#### ZFNs

ZFNs are site-specific custom-designed endonucleases that act through a combination between zinc finger proteins, which direct the gene-editing event to predetermined DNA sequences, and *Fok*I DNA restriction enzymes, which introduce cleavages into the intended DNA sequences (Urnov et al., 2010). ZFNs apply the same principle of mediating site-specific modifications *via* induction of DSB repair pathways (**Figures 2** and **3**). ZFNs are considered as the first “practical” DSB-assisted gene-editing tool that has been applied for the introduction of desired manipulations to cell lines as well as to organisms in a relatively easier way than the previously reported meganucleases. Cleaving the target DNA can be achieved by binding and aligning two ZFN monomers to their corresponding DNA target sequences in a tail-to-tail orientation (Weinthal et al., 2010). Each ZFN monomer is composed of a ZF domain (DNA-binding domain) and a non-specific *Fok*I domain (DNA-cleavage domain). Typically, the ZF domain is composed of three to four individual fingers, each being capable to recognize and bind to an approximately 3-bp-long sequence (triplet bp within the DNA substrate) (Wu et al., 2007; Weinthal et al., 2010). This means that the ZF domain of three or four individual fingers can recognize and bind to DNA sequences with lengths of 9 or 12 bp.

ZFNs have been applied to farm animals including sheep and goats. In sheep, ZFNs have been applied to target the *MSTN* gene in fetal fibroblasts (Zhang et al., 2014a; Zhang et al., 2016b), primary satellite cells (Salabi et al., 2014), and embryos (Zhang et al., 2016b). The results of these reports indicated the potential of ZFNs to introduce gene disruption in both *MSTN* exon 1 (Zhang et al., 2014a), and exon 3 (Salabi et al., 2014; Zhang et al., 2016b) with both mono- and bi-allelic knockouts. Cytoplasmic injection of *MSTN*-ZFN mRNA into ovine embryos at the one-cell stage achieved 35% (13/37) efficiency (Zhang et al., 2016b). These studies highlight the potential of ZFNs to generate *MSTN* gene knockout founders using somatic cloning or ZFN microinjection into embryos (**Figure 3B**).

In addition to targeting *MSTN* to promote the growth and muscle mass in meat-producing lambs, *BLG*, a dominant allergen in milk, has also been targeted with desirable outcomes in dairy goats. ZFNs have been designed to mediate disruption in the goat *BLG* gene. Different methods have been used to deliver ZFNs that specifically target *BLG* in goat fibroblasts, including transfection of ZFN-expressing plasmid (Xiong et al., 2013), direct delivery of ZFNs as purified proteins (Song et al., 2015), and electroporation of ZFN-expressing plasmid (Yuan et al., 2016). Using purified ZFN proteins for targeting has potential to reduce insertional mutagenesis, toxicity, and off-target events (Gaj et al., 2012; Song et al., 2015). In general, the results of these initial studies have corroborated the ability of ZFNs to mediate *BLG* disruption in goats. Another interesting example for the application of ZFNs in goats includes the generation of gene knockout fetuses by ZFN-mRNA cytoplasmic injection into fertilized oocytes. For this, ZFNs have been designed to introduce biallelic disruption in *forkhead box L2 (FOXL2)* to investigate the function of this gene in female sex determination (Boulanger et al., 2014).

Although, no reports indicate the production of live gene-edited sheep and goats using ZFNs, the published data on the use of ZFNs to target cell lines, embryos, and fetuses indicate their potential ability for the generation of live genetically modified sheep and goats. However, the further emergence of simpler tools such as TALENs and CRISPRs has facilitated the application of gene editing for the generation of live founders with intended genomic modifications.

#### TALENs

TALENs are customizable DNA nucleases that have rapidly emerged as a desirable alternative for ZFNs with the ability to mediate site-specific modifications based on the principle of introducing DSBs (Bogdanove and Voytas, 2011; Joung and Sander, 2013) (**Figures 2** and **3**). TALENs resemble ZFNs in which a nonspecific *Fok*I DNA-cleaving domain is fused to a customizable DNA-binding domain to generate functional DSB-introducing nucleases. TALEs are naturally occurring proteins that are secreted by plant pathogenic bacteria *Xanthomonas* species. These can cause disease in plants after injection into host cells and *via* interference with cellular activities by activating the transcription of specific target genes (Chen and Gao, 2013). TALENs function as dimers, and each DNA-binding domain is composed of a series of tandem repeats, each of which comprises 33–35 amino acids that can recognize and specifically bind to a single DNA nucleotide (Sun and Zhao, 2013). Each TALEN is designed to bind to ∼20 nucleotides with a DNA spacer consisting of ∼14–20 nucleotides between both TALEN dimers. This forms a range of ∼54–60 nucleotides for recognition and targeting (Sanjana et al., 2012). ZFNs and TALENs cleave DNA with relatively similar efficiency; however, the main advantage of TALENs over ZFNs is that TALENs are easier for design and construction (Joung and Sander, 2013). The simplicity of TALENs over ZFNs has extended the application of gene editing and facilitated the generation of organisms as well as cell lines with specific genetic alterations.

TALENs have been applied to sheep and goats and live founders with desired genetic alterations have been generated. In sheep, TALENs have been used to generate *MSTN*-knockout lambs. One out of nine live births produced using TALEN-mRNA cytoplasmic injection was *MSTN*-edited (Proudfoot et al., 2015). TALENs combined with single-stranded oligodeoxynucleotides (ssODNs) carrying a stop codon to target exon 2 of the *MSTN* gene have been transfected to sheep primary fibroblasts; 11.4% (4/35) of the sequenced colonies contained the desired insertion (Zhao et al., 2016). Fibroblasts with modified *MSNT* were used as nuclear donor for SCNT. After full-term gestation, one lamb was born and died soon after birth. DNA sequencing of tissues from the cloned lamb showed identical insertion of a stop codon site in the *MSTN* gene with donor cells (Zhao et al., 2016). In a further report, TALEN-mediated *MSTN* biallelic-knockout somatic cells were used as nuclear donor cells for SCNT (Li et al., 2016). 16 out of 23 lambs were obtained (12 live and 11 dead) that showed expected biallelic mutations of the *MSTN* gene. The live founders showed a remarkable increase in body weight compared to their wild-type counterparts (Li et al., 2016).

In goats, TALENs were first reported in fibroblasts to introgress SNP alleles that are responsible for fecundity in sheep (*bone morphogenetic protein receptor type 1B, BMPR-IB;* also known as *Booroola fecundity, FecB)* and muscle hypertrophy *(callipyge, CLPG)* into the goat genome using TALEN mRNA and oligonucleotide transfection (Tan et al., 2013). This initial report presented the potential of the TALEN endonucleases for the introduction of desirable allelic introgressions into the genomes of farm animals. TALENs have also been applied in goats to target the *BLG* gene (Cui et al., 2015; Ge et al., 2016; Zhu et al., 2016b; Yuan et al., 2017). Furthermore, gene-edited goats that carry both *BLG* knockout and enriched expression of *human lactoferrin (hLF)* (Cui et al., 2015) or *human α-lactalbumin (hLA)* (Zhu et al., 2016b) have been generated using SCNT. Cui et al. performed two rounds of cloning to generate *BLG* biallelic knockout goats with an enriched expression of *hLF* in milk. 10 kids were generated during the first round of cloning (seven *BLG*^+/-^ and three *BLG*^+/hLF^), and after the second targeting and cloning, five cloned kids were generated (three *BLG*^-/-^ and two *BLG*^-/hLF^) (Cui et al., 2015). Furthermore, Zhu et al. reported the generation of *BLG* knockout goats with enriched expression of *hLA* in milk, six live births were obtained, one was a biallelic targeted goat (which died after 1 month), and the other five goats were *BLG*^hLA/+^. *BLG* expression in the milk of transgenic goats was reduced, while *hLA* was highly expressed compared to normal goats (Zhu et al., 2016b). Caprine *MSTN* knockout using TALENs has also been reported. Three cloned kids have been produced, two have died after birth (*MSTN*^-/-^ and *MSTN*^+/+^), and one was alive and healthy (*MSTN*^+/-^) (Yu et al., 2016). TALENs have contributed to the generation of live and genetically altered sheep and goats with desired phenotypes; however, the procedure of gene editing has to become simpler and quicker with more advanced modification systems.

#### CRISPR/Cas9

Rapid and promising advances have been reported in the field of genetic modification during the past decade. The revolution of genetic engineering has culminated in the emergence of CRISPR systems. These are simple but sophisticated mechanisms derived from nature that act in prokaryotes as an adaptive immune system against phage and foreign DNA infection by the cooperation of CRISPR sequences with Cas proteins (Mojica and Montoliu, 2016). Based on many years of research, CRISPR systems were developed from prokaryotic adaptive defense systems to robust gene-editing tools applicable throughout the entire biological kingdom (Doudna and Charpentier, 2014).

CRISPR systems have attracted scientific attention after their role as simple, precise, and efficient nucleases that can introduce DSBs within DNA sequences in a site-specific manner has been highlighted. Of the three types of CRISPR/Cas systems, type II *Streptococcus pyogenes* CRISPR-Cas9 is the most widely used CRISPR system. The CRISPR/Cas9 system uses two main components: an RNA-directed Cas9 protein and ∼20-nucleotide sgRNA, which leads the Cas9 protein to a user-defined DNA target site as long as it is next to a protospacer adjacent motif (PAM) sequence (Doudna and Charpentier, 2014). PAM is a short sequence within the targeted DNA that acts as a recognition site. Introducing DSBs into the targeted genomes stimulates various forms of gene-editing events based on the natural DNA repair ability (Gaj et al., 2013) (**Figure 3C**). The CRISPR/Cas9 system differs from ZFNs and TALENs since it is an RNA-directed system based on the Cas9 protein that introduces DSBs instead of protein-directed *Fok*I restriction endonuclease in both ZFNs and TALENs. One of the main advantages of directing the nuclease *via* RNA is the simple construction of RNA-expressing constructs and thus the expansion of the ability to direct the nuclease to any desired target sequence.

CRISPR systems were first applied to mammalian genomes in 2013 (Cong et al., 2013; Mali et al., 2013); during the same year, CRISPR systems have been applied to generate mutant mice with a number of modifications (Shen et al., 2013; Wang et al., 2013; Yang et al., 2013). Consequently, CRISPR systems have been applied to a wide range of cell lines as well as living organisms. Recently, new CRISPR systems have been discovered, thus extending the toolbox of gene editing with further options for efficient and precise targeting and/or manipulation (Komor et al., 2017). These systems include catalytically inactive/dead Cas9 (dCas9), which has been employed in gene regulation, epigenetic modification, chromatin engineering, and base editing (reviewed by Adli, 2018). Single-nucleotide alterations have been applied using base editor systems composed of dCas9, and further versions have been developed using Cas9 nickase (nCas9) (Komor et al., 2016; Eid et al., 2018). These enable new and more precise forms of genomic modifications. Due to their simplicity, affordability, and customizability, CRISPR systems (especially Cas9-based systems), have initiated a great biotechnological revolution in different fields including basic research, biomedicine, and agriculture.

In general, despite the advantages and disadvantages of the above-mentioned genetic modification techniques (see **Table 7**), all of these have significantly increased our knowledge of the nature of ovine and caprine genomes. Furthermore, they enabled the generation of a large variety of useful, genetically manipulated sheep and goat models.

**Table 7:** The advantages and disadvantages of genetic manipulation tools applicable to ovine and caprine genomes

Tool	Uses	Advantages	Disadvantages	References
PNI	Insertional transgenesis	The first tool to be applied for the generation of transgenic animals	Random integration, variable transgene copy number, low efficiency	Hammer et al., 1985; Clark, 2002
SCNT	Gene targeting, editing	An alternative that facilitated the implementation of HR gene targeting in species that lack ESCs, a low-level mosaicism	Development of a small proportion of reconstructed embryos that become live offspring, potential complications at birth of offspring as a result of developmental abnormalities	Schnieke et al., 1997; Wilmut et al., 1997; Wilmut et al., 1999
SMGT	Gene transfer, integration	Simple, cost-effective, minimal embryo handling required	Initial doubt with regard to its repeatability, variable results, low incorporation of the exogenous gene	Lavitrano et al., 1989; Wall, 2002; Lavitrano et al., 2006
VMGT	Gene transfer, integration	Able to infect germline cells and dividing or non-dividing somatic cells, delivery of the system to the egg/zygote is less damaging compared to pronuclear injection, high integration	Variability of transgenic expression, potential health risks, limited DNA capacity	Whitelaw et al., 2008; Modric and Mergia, 2009
Recombinases	Integration, selectable cassette excision	Increased gene integration efficiency, offer different forms of modifications including the removal of unwanted DNA	Conservative specificity, in specific cases, pre-introduction of specific target sites within the host genome is required which is an inefficient and time-consuming process, potential toxicity	Xu et al., 2008; Gaj et al., 2014; Olorunniji et al., 2016
Transposons	Integration	Able to integrate transgenes and RNAi-expressing constructs for the mediation of knockdown expression, lower immunogenicity and larger DNA capacity compared to viral systems	Classical transposons are less efficient for gene transfer compared to viral systems, potential cytotoxicity	Muñoz-López and García-Pérez, 2010; Meir and Wu, 2011; Hudecek et al., 2017
RNAi	Gene knock down	Targeting gene expression at mRNA level, useful tool to elucidate gene functions	Variability and incompleteness of knockdowns, potential off-target	Boutros and Ahringer, 2008; Boettcher and McManus, 2015; Bradford et al., 2017
ZFNs	Gene editing	First “practical” endonuclease that has been applied to mediated gene-editing events	Difficult to design, potential off-target, mosaicism in offspring generated from microinjected embryos	Gaj et al., 2013; Gupta and Musunuru, 2014; Oliver et al., 2015; Zhang et al., 2019a
TALENs	Gene editing	A simplified alternative of the previously emerged ZFNs	Moderate difficulty in design, potential off-target, mosaicism in offspring generated from microinjected embryos	Bedell et al., 2012; Gaj et al., 2013; Gupta and Musunuru, 2014; Zhang et al., 2019a
CRISPR/Cas9	Gene editing	Simple, cost-effective, customizable, precise compared to other endonucleases, able to mediate multiplex editing	Potential off-target, mosaicism in offspring generated from microinjected embryos	Gaj et al., 2013; and Gupta and Musunuru, 2014; Mehravar et al., 2019; Zhang et al., 2019a

PNI, pronuclear injection; SCNT, somatic cell nuclear transfer; SMGT, sperm-mediated gene transfer; VMGT, virus-mediated gene transfer; RNAi, RNA interference; ZFNs, zinc finger nucleases; TALENs, transcription activator-like effector nucleases; CRISPR/Cas9, clustered regularly interspaced short palindromic repeat/CRISPR-associated protein 9.

## Applications of CRISPR/Cas9 in Sheep and Goats

Gene editing has been revolutionized as a result of the rapid emergence of novel varieties of tools that can simply, precisely, and more efficiently mediate different forms of DNA modifications than previously reported tools. CRISPR/Cas9 systems have been applied to sheep and goats to fulfill various promising purposes (**Figure 1**). To date, a number of sheep and goat models have been generated *via* CRISPR/Cas9 systems (**Table 3**). Moreover, further studies are ongoing for the provision of useful sheep and goat models for agriculture and biomedicine. The following paragraphs outline the applications of CRISPR/Cas9 in sheep and goats.

### Promotion of Muscle Growth and Development

Increasing the body weight and accelerating the growth rates of farm animals are important aims in agriculture. Genes that affect these traits are attractive targets for emerging disruptive gene-editing techniques. *MSTN* was among the first genes that have been subjected to CRISPR/Cas9 targeting, as a strategy to achieve an economically important trait by applying gene-editing tools in sheep and goats. CRISPR/Cas9 targeting of *MSTN* was first applied in sheep, where 35 founders were obtained, two of which contained the mutation (5.7%) (Han et al., 2014). Later, Crispo et al. reported the production of *MSTN*-disrupted sheep using CRISPR/Cas9, where 10 lambs out of 22 obtained founders (45.4%) showed the mutation with heavier body weight compared to their wild-type counterparts (Crispo et al., 2015a). These initial reports encouraged the further application of CRISPR/Cas9 systems in small ruminants.

In our team, CRISPR/Cas9 has been applied to achieve multiplex gene editing of *MSTN* with two other economically important genes, including the *agouti-signaling protein (ASIP)* and *β-carotene oxygenase 2 (BCO2)* in sheep (Wang et al., 2016b). 49 founders have been obtained, 36 of which were alive. Among these 36 live lambs, the targeting efficiencies were 27.7% (10/36) for *MSTN*, 33.3% (12/36) for *ASIP*, and 27.7% (10/36) for *BCO2*; 5.6% (2/36) showed the simultaneous targeting of all three genes. No off-target has been detected and founders with *MSTN* mutations showed enlarged myofibers and enhanced body weight compared to wild-type individuals (Wang et al., 2016b). In addition, sheep with biallelic modification in the *BCO2* gene showed yellow fat compared to the white fat color of monoallelic and wild-type individuals, highlighting the role of *BCO2* in the fat color determination in sheep (Niu et al., 2017). To ensuring the biosafety of CRISPR/Cas9 in large animals, further steps have been taken by performing trio-based whole genome sequencing (for the edits and their parents) to investigate the origins of the variations in the generated edits, which might be parentally inherited, naturally obtained, or induced by a specific targeting event (Wang et al., 2018a). The results that were obtained from the multiplex edited sheep showed negligible off-target modifications that did not affect the application of CRISPR/Cas9 in large animals. In summary, these results highlight the potential of the CRISPR/Cas9 system to introduce multiplex editing in farm animals.

Moreover, disrupting the normal function of *MSTN* in sheep skeletal muscle satellite cells (sSMSCs) has been shown to promote sSMSC differentiation in both number and length. This study has also reported the generation of *MSTN*-disputed sheep using SCNT from CRISPR/Cas9 transfected ear fibroblasts (Zhang et al., 2018c). A further and more recent report, published by our team, described the application of CRISPR/Cas9-based base editors for the introduction of a point mutation within the *suppressor of cytokine signaling 2 (SOCS2)* gene in sheep (Zhou et al., 2019). This single-nucleotide variant exerts profound effects on both body weight and size as well as milk production. This study highlights the potential role of base editors in sheep and goats, which can be utilized to introduce single alterations of bases that harbor desirable economical traits.

In goats, CRISPR/Cas9 was used in a study that targeted the four important genes, *MSTN*, *BLG*, *PrP,* and *nucleoporin 155 (NUP155)* in goat fibroblasts and generated three *MSTN* knockout goats using SCNT (Ni et al., 2014). The reported efficiencies of CRISPR/Cas9 in goat fibroblasts ranged from 9 and 70%, indicating the ability of CRISPR/Cas9 to efficiently work in the caprine system. Later, gene-modified goats that carry knockouts either in *MSTN* or *fibroblast growth factor 5 (FGF5)* or both genes have been reported by our team (Wang et al., 2015). Of 98 obtained individuals (including 79 delivered alive, 14 delivered but died shortly after birth, and five aborted), 15/98 (15.3%) carried a disruption in *MSTN*, 21/98 (21.4%) carried a disruption in *FGF5*, and 10/98 (10.2%) showed simultaneous disruption of both genes. These results confirm the efficient induction of multiplex targeting *via* CRISPR/Cas9, which is of great importance in farm animals. Especially, since most of the economically important traits are controlled by multiple loci. Further studies have been conducted that used the *MSTN* mutated founders generated from this experiment to confirm the occurrence of gene disruption and the transmission of the knockout alleles (Wang et al., 2018b), as well as to analyze the transcriptomic changes of *MSTN* knockout goats (Wang et al., 2017). The occurrence and transmission of editing events have been confirmed, and substantial changes in gene expressions have been determined at the transcriptome level. These expressional changes were found in genes that are involved in fatty acid metabolism and unsaturated fatty acid biosynthesis, suggesting a regulatory role of *MSTN* in the expression of these genes. Furthermore, family trio-based deep sequencing for gene-edited goats and their progenies was performed to investigate the occurrence of *de novo* mutations, indels, and other structural variants (Li et al., 2018a). The obtained results of this report support the reliability of CRISPR/Cas9 application in large animals.

Further publications have also reported the generation of *MSTN* knockout sheep and goats *via* the CRISPR/Cas9 system (see **Table 3**). Among these, an interesting report described the generation of a goat kid carrying simultaneous *MSTN* knockout and *fat1* knockin using CRISPR/Cas9 combined with SCNT (Zhang et al., 2018a). The efficiency of simultaneous targeting was 25.6% (40/156) in goat fibroblasts. Despite this moderate efficiency at the cellular level, one edited founder out of 134 transferred cloned embryos was generated. This ratio might be increased by improving SCNT conditions. The efficiencies of TALEN and CRISPR/Cas9 for the targeting of caprine *MSTN* have also been compared. Despite several advantages of the former endonuclease, the latter has shown a higher generation frequency of biallelic disruptions and longer deletions (Zhang et al., 2019b). CRISPR/Cas9 has significantly highlighted the functional role of genes related to muscle growth and body weight both in sheep and goats, further emphasizing the potential role of gene editing to provide the farm animal sector with novel breeds that carry desirable and valuable traits.

### Promotion of Fiber Length and Growth

Sheep and goats form a valuable source for the production of fibers. Genes associated with fiber quality and quantity are a source of attraction, and many researchers hope to alter these genes in attempts to obtain desired and new fiber properties. The *FGF5* gene, which is a dominant inhibitor of fiber length and growth, is an attractive target. In sheep, CRISPR/Cas9 has been applied to disrupt the normal function of the *FGF5* gene, resulting in 3/18 (16.6%) mutated founders that carried a disruption in *FGF5* and showed increased wool length (Hu et al., 2017). Another publication has also reported the generation of *FGF5*-disrupted sheep; 16/20 (80%) mutated founders carried both monoallelic and biallelic mutations in *FGF5* and showed increased wool length and quantity (Li et al., 2017c). Recently, Zhang et al. have also confirmed that the disruption of *FGF5* in sheep can lead to an increased wool length and average wool growth rate (Zhang et al., 2019c). The results of these studies confirm the functional role of *FGF5* and its desired disrupting effect. In an interesting study related to fiber characteristics, Zhang et al. introduced the targeted disruption of the *ASIP* gene by using CRISPR/Cas9 (Zhang et al., 2017a). The resultant founders that carried disruption within *ASIP* have shown various coat color patterns versus the white coat color of wild-type individuals of the same breed (the Chinese merino). This highlights the critical role of the *ASIP* gene in coat color determination in sheep.

In the *MSTN/FGF5* knockout goat model (Wang et al., 2015), further confirmation of the gene-editing event of the resultant founders has been performed. The simultaneous occurrence of *FGF5* disruption at both the morphological and genetic levels has been confirmed and an enhancement in fiber length, as well as an increase in the number of secondary hair follicles were obtained (Wang et al., 2016a). CRISPR/Cas9 has also been used to generate *ectodysplasin receptor (EDAR)* gene knockout goats by SCNT to investigate the disruption effects on the phenotype, hair follicle growth and development (Hao et al., 2018). *EDAR*-knockout founders showed abnormal primary hair follicles and an absence of hair on the top of their heads; these characteristics are distinctive features of *EDAR* mutants. These generated founders provide a useful model for the study of the relationship between the *EDAR* gene and hair follicle growth and development. Investigating the functional roles of genes that are directly associated with hair growth and development is of great importance in wool- and cashmere-producing breeds. Recently, CRISPR-mediated base editing has been applied by our team to introduce nonsense codon introgression into the *FGF5* gene to enhance the yield of cashmere hair in goats (Li et al., 2018b). Five newborns out of 22 transferred embryos were generated that carried at least one nonsense mutation or other mutational types that were induced by the base editing system. In addition to our recent report regarding the applications of base editing in sheep (Zhou et al., 2019), these results highlight the potential of base editor application to the genomes of farm animals to obtain desirable economical traits that are harbored by single bases. CRISPR/Cas9 has shown potential for disrupting genes that inhibit fiber desirable phenotypes in sheep and goats, which establishes a new platform to rapidly achieve the aims of animal breeding based on gene editing.

### Molecular Manipulation of Milk Components

Manipulation of milk components and the expression of desired transgenes in milk with the aim to enrich its components with valuable proteins are among the main aims in livestock genetic modification programs. CRISPR/Cas9 has been applied in sheep and goats to alter the characteristics of milk by inhibiting the expression of undesired proteins or by applying gene knockin strategies to enrich the milk with new desired expression products. Initially, CRISPR/Cas9 has been used to target *BLG* in goat primary fibroblasts (19%) (Ni et al., 2014), and later, gene-edited goats with disrupted *BLG* gene have been generated (Zhou et al., 2017). Four founders out of 26 (15.38%) were *BLG* edits, which have shown decreased expression of *BLG* and abolished BLG protein production in milk (Zhou et al., 2017). The generated founders of this study provide a useful model for the study of the relationship between BLG and other milk proteins. Furthermore, this study provides a useful caprine model that can generate BLG-free milk.

Induction of genetic integration into the genomes is of great interest, especially for the introduction of new expressional characteristics in milk. CRISPR/Cas9 has shown the ability to introduce knockin of the marker gene *turbo GFP (tGFP)* in the *Rosa26* locus of sheep genome (Wu et al., 2016). This study also indicates the *Rosa26* locus as a potential site for exogenous gene expression in sheep. Another example of CRISPR/Cas9 application for the manipulation of milk components includes the generation of gene-edited sheep with an enriched production of melatonin in milk (Ma et al., 2017). In this approach, Cas9 mRNA, sgRNA, and the linearized vectors carrying *arylalkylamine N-acetyltransferase (AANAT)* and *acetylserotonin methyltransferase (ASMT)* for the expression of melatonin were cytoplasmically co-injected into pronuclear embryos. Of 34 transgenic founders, seven carried *AANAT*, two carried *ASMT*, and 25 carried both of *AANAT* and *ASMT* genes. Founders carrying these genes produced melatonin-enriched milk. These models might be a good source of melatonin, which has various nutritional and medicinal values and uses.

CRISPR/Cas9 has also been used to investigate the functional role of genes such as *stearoyl-CoA desaturase 1 (SCD1)* in goat mammary epithelial cells (Tian et al., 2018a) and *acetyl CoA acyltransferase 2 (ACAA2)* in sheep precursor adipocyte cells (Zhang et al., 2019d). These genes are directly or indirectly related to milk traits and affect the fatty acid metabolism. CRISPR/Cas9 has facilitated the generation of gene-modified sheep and goats with specific milk characteristics which might also facilitate the large-scale production of useful proteins and pharmaceuticals in milk.

### Promotion of Reproductive Performance

Improving the reproductive performance is an important direction of livestock breeding. Desirable traits related to reproductivity such as litter size have been suggested as goals for introduction to farm animals using gene-editing tools instead of the comparatively tedious conventional breeding strategies. Mutations in the sheep *BMPR-IB* *(FecB)* gene has shown to be responsible on an increased ovulation rate and consequently larger litter size (Fabre et al., 2006). Initially, CRISPR/Cas9 has been reported to target sheep *BMPR-IB* *in vitro*, resulting in gene-edited embryos that can be characterized by a variety of indels at the *BMPR-IB/FecB* locus (Zhang et al., 2017b). Sheep *BMPR-1B* has also been targeted by our team using an ssODN-based approach to introduce defined point mutations. Gene-edited founders have been produced. Seven out of 21 delivered lambs contained editing events, five of which have been determined to carry the intended nucleotide substitution (Zhou et al., 2018). The targeting efficiency was 33.3% (7/21) in the generated founders, and the efficiency of the intended single-nucleotide substitution was 23.8% (5/21).

In goats, another defined point mutation has also been introduced by our team in the *growth differentiation factor 9 (GDF9)* gene, which exerts a large effect on both ovulation rate and litter size (Niu et al., 2018). In this study, four out of 18 delivered kids carried the intended mutation (22.2% targeting efficiency). In summary, the results of both reports highlight the role of CRISPR/Cas9-induced HDR with an ssODN template for introducing reliable and defined point mutations in livestock. In another experiment, the effects of open pulled straw (OPS) vitrification as a method to preserve microinjected embryos on *AANAT*-microinjected embryo development and the reproductive capacity of produced *AANAT*-transgenic offspring have been investigated (Tian et al., 2018b). In this study, a number of both frozen and non-frozen microinjected sheep embryos have been used to compare and generate live transgenic offspring. The results of this study showed no significant differences between the frozen and non-frozen *AANAT*-microinjected embryos. Furthermore, *AANAT*-transgenic individuals have shown improved reproductive capacity. CRISPR/Cas9 has also been applied to investigate the biological role of the *glucocorticoid receptor (NR3C1)* in sheep conceptus elongation using recovered elongating conceptuses (Brooks et al., 2015). However, the study highlighted the essential roles of other factors rather than *NR3C1* in conceptus elongation in sheep. CRISPR/Cas9 has been applied to develop desirable traits for the improvement of the reproductive performance of sheep and goats.

### Generation of Disease-Resistant Animals

Gene editing has been suggested as a robust tool that can be used to generate disease-resistant animals. Consequently, genetically modified resistant farm animals have been produced. These offer good models to investigate and understand disease pathogenesis and offer a potential source for the spread of disease resistance traits in commercial flocks. The CRISPR/Cas9 system has been applied in sheep and goats to achieve aims related to improving animal health and welfare. Several disease-related genes have been disrupted using CRISPR/Cas9. The *PrPc* is directly associated with the pathogenesis of the transmissible spongiform encephalopathies, which occur in humans and a number of livestock species including sheep and goats (Yu et al., 2006). *PrP*-resistant animals can be produced by suppressing the expression of *PrP* (Golding et al., 2006). CRISPR/Cas9 has been applied to target *PrP* in goat fibroblasts with the aim to generate *PrP*-knockout donor cells that can be used in SCNT for the production of *PrP*-resistant goats (Ni et al., 2014; Hu et al., 2015; Fan et al., 2019). The targeting efficiency of *PrP* increased by 70% in goat fibroblasts and by 20% (9/45) for the simultaneous targeting of *PrP* and *MSTN*. Moreover, of the nine dual gene mutant colonies, five had mutations in all four alleles of both genes. These results suggest the use of the CRISPR/Cas9 system to target genes to confer potential disease resistance in farm animals.

Menchaca et al. indicated the application of the CRISPR/Cas9 system to induce the loss of function of *hyaluronidase 2 (HYAL2)*, which can be used by Jaagsiekte sheep retrovirus as a cell entry receptor, resulting in ovine pulmonary adenocarcinoma syndrome (Menchaca et al., 2018). As indicated by the authors, generation of *HYAL2*-knockout lambs will validate the possibility to apply the CRISPR/Cas9 system for producing virus-resistant farm animals and will help to investigate the functional roles of *HYAL2*. It seems that the research of genetic engineering via CRISPR systems for the generation of disease-resistant sheep and goats is ongoing, and maybe varieties of genetically modified resistant sheep and goats will be reported soon, thus enriching the field with novel and valuable models.

### Generation of Models for Human Diseases

Sheep and goats have been used as interesting models in biomedical research. Compared to experimental rodents, sheep and goats offer the advantage of being more suitable mimics for human diseases due to their similar size and anatomy. CRISPR/Cas9 has been applied in sheep and goats to provide biomedical research with useful models to investigate human diseases. In mice, mutations within *NUP155* were associated with atrial fibrillation and early sudden cardiac death (Zhang et al., 2008b). Large *NUP155*-knockout animals could be useful models for research in cardiac physiology. Initially, CRISPR/Cas9 has been applied to target the *NUP155* gene in goat fibroblasts to generate *NUP155*-knockout donor cells, which can be used in the SCNT program to produce *NUP155*-knockout goat model (Hu et al., 2014; Ni et al., 2014; Fan et al., 2019).

In humans, during pregnancy, Zika virus (ZIKV) infection can lead to fetal infection, causing microcephaly and other severe congenital neurological symptoms. Type I interferons (IFNs) are central for host resistance against ZIKV, and *interferon α/β receptor IFNAR*-deficient mice have been shown to be highly susceptible to ZIKV infection (Yockey et al., 2018). Fan et al. reported the generation of *IFNAR*-knockout sheep by applying CRISPR/Cas9 in combination with SCNT to provide highly susceptible ZIKV large animal model (Fan et al., 2017).

Fan et al. have also provided an interesting sheep model for the investigation of human cystic fibrosis (CF) by employing both CRISPR/Cas9 and SCNT (Fan et al., 2018). CF is an inherited disorder that affects mostly the lungs and other organs in the body. The authors have introduced targeting of the *CF transmembrane conductance regulator (CFTR)* gene and *CFTR*^–/–^ as well as *CFTR*^+/–^ lambs have been produced with a severe phenotype of CF pathology similar to that of humans.

Additionally, Williams et al. have also reported an interesting sheep model, recapitulating human hypophosphatasia (HPP; a rare metabolic bone disease) by applying CRISPR/Cas9 (Williams et al., 2018). In this study, a single point mutation in the tissue-nonspecific alkaline phosphatase (TNSALP) gene *(ALPL, alkaline phosphatase, biomineralization associated)* has been introduced. The thus generated gene-edited lambs accurately phenocopied human HPP, providing a useful large animal model for the study of rare human bone diseases. The results of these reports corroborate the great potential of the CRISPR/Cas9 system to generate gene-edited sheep and goats that recapitulate human diseases.

### Xenotransplantation and Generation of Hosts for the Growth of Human Organs

One of the most promising strategies to solve the shortage of organ donors worldwide is to generate human organs inside large animals by applying a technique called interspecies blastocyst complementation (De Los Angeles et al., 2018). In this technique, large animals act as hosts and grow human organs. The procedure of this technique is based on a combination of gene-edited embryos of large animals and human pluripotent stem cells (PSCs). In this case, gene-editing tools are used to generate large animal embryos with genetically engineered “organ niches” by disabling a specific gene or a number of genes that are responsible for the formation of a target organ, thus allowing human PSCs to colonize the vacant niche and generate the desired organ “gest-derived organ” (Rashid et al., 2014).

In this context, an interesting study reported the creation of pancreatogenesis-disabled sheep as a step toward achieving interspecies blastocyst complementation–based xenotransplantation between human and large animals (Vilarino et al., 2017; Vilarino et al., 2018). In this experiment, CRISPR/Cas9 has been applied to target *pancreatic and duodenal homeobox protein 1 (PDX1),* which is a necessary gene for pancreatic development. CRISPR/Cas9 combined with dual sgRNAs and direct oocyte microinjection approach was used to generate *PDX1* disruption, and a resulting fetus with *PDX1*^-,-^ mutation lacked a pancreas. The results of these promising attempts highlight the potential of gene-edited sheep to be applied in interspecies organ generation as candidate hosts for the growth of human organs. Integrative programs that employ CRISPR/Cas9 and PSC complementation offer a potential solution for the shortage of organ donors.

## Conclusions and Future Perspectives

In the current era, genome engineering systems have become indispensable tools of basic research, biomedicine, and agriculture. Genome engineering tools, especially the recently emerged CRISPR systems, offer the potential to revolutionize all biological research fields including research on agriculture and farm animals. Applying genome engineering tools to farm animals including sheep and goats is of significant importance. Currently, gene-edited sheep and goats are generated with relative ease using CRISPR systems, providing valuable models for agricultural, veterinary, and biomedical research. The list of sheep and goat genes targeted by CRISPR systems is continuously growing. In the future, a large number of genes will be targeted, and more valuable models will be generated, thus widening our understanding about these genes and their corresponding phenotypes and functions. This basic research is only the beginning; the potent potential of genome engineering will become apparent in the future.

## Author Contributions

All authors listed have made a substantial, direct and intellectual contribution to the work, and approved it for publication.

## Funding

This study was supported by the National Natural Science Foundation of China (31772571, 31572369, 31872332), and Local Grants (NXTS2018-001, 2017NY-072, and 2018KJXX-009). Xiaolong Wang is a Tang Scholar at Northwest A&F University, China.

## Conflict of Interest Statement

Author TS was employed by Recombinetics Inc. The remaining authors declare that the research was conducted in the absence of any commercial or financial relationships that could be construed as a potential conflict of interest.
